# Blood-feeding ecology of mosquitoes in two zoological gardens in the United Kingdom

**DOI:** 10.1186/s13071-021-04735-0

**Published:** 2021-05-21

**Authors:** Arturo Hernandez-Colina, Merit Gonzalez-Olvera, Emily Lomax, Freya Townsend, Amber Maddox, Jenny C. Hesson, Kenneth Sherlock, Dawn Ward, Lindsay Eckley, Mark Vercoe, Javier Lopez, Matthew Baylis

**Affiliations:** 1 Department of Livestock and One Health, Institute of Infection, Veterinary & Ecological Sciences, University of Liverpool, Leahurst Campus, Chester High Road, Neston, Cheshire, CH64 7TE UK; 2 North of England Zoological Society (Chester Zoo), Caughall Road, Chester, CH2 1LH UK; 3 Department of Medical Biochemistry and Microbiology, Zoonosis Science Centre, Uppsala University, 751 23 Uppsala, Sweden; 4 Flamingo Land, Kirby Misperton, Malton, YO17 6UX UK; 5 Health Protection Research Unit in Emerging and Zoonotic Infections, University of Liverpool, Liverpool, UK

**Keywords:** Blood meal, *Culex pipiens*, *Culiseta annulata*, Mosquito control, Mosquito dispersal

## Abstract

**Background:**

Zoological gardens contain unique configurations of exotic and endemic animals and plants that create a diverse range of developing sites and potential sources of blood meals for local mosquitoes. This may imply unusual interspecific pathogen transmission risks involving zoo vertebrates, like avian malaria to captive penguins. Understanding mosquito ecology and host feeding patterns is necessary to improve mosquito control and disease prevention measures in these environments.

**Methods:**

Mosquito sampling took place in Chester Zoo for 3years (2017, 2018, and 2019) and for 1year in Flamingo Land (2017) using different trapping methods. Blood-fed mosquitoes were identified and their blood meal was amplified by PCR, sequenced, and blasted for host species identification.

**Results:**

In total, 640 blood-fed mosquitoes were collected [*Culex pipiens* (*n*=497), *Culiseta annulata* (*n*=81), *Anopheles maculipennis* s.l. (*n*=7), *An. claviger* (*n*=1), and unidentifiable (*n*=55)]. Successful identification of the host species was achieved from 159 blood-fed mosquitoes. Mosquitoes fed on birds (*n*=74), non-human mammals (*n*=20), and humans (*n*=71). There were mixed blood meals from two hosts (*n*=6). The proportions of blood-fed mosquitoes varied across sampling seasons and sites within the zoos. The use of resting traps and aspiration of vegetation were more efficient techniques for capturing blood-fed mosquitoes than traps for host-seeking or gravid mosquitoes. By relating the locations of zoo vertebrates to where fed mosquitoes were trapped, the minimum travelling distances were calculated (13.7 to 366.7m). Temperature, precipitation, relative humidity, proximity to zoo vertebrate exhibits, and vegetation level were found to be significantly associated with the proportion of captured blood-fed mosquitoes by generalized linear modelling.

**Conclusions:**

Mosquito feeding behaviour in zoos is mainly influenced by time, location (sampling area), temperature, and host availability, which highlights the value of mosquito monitoring in complex settings to plan control strategies and potentially reduce inherent disease transmission risks for humans and threatened zoo vertebrates.

**Graphic abstract:**

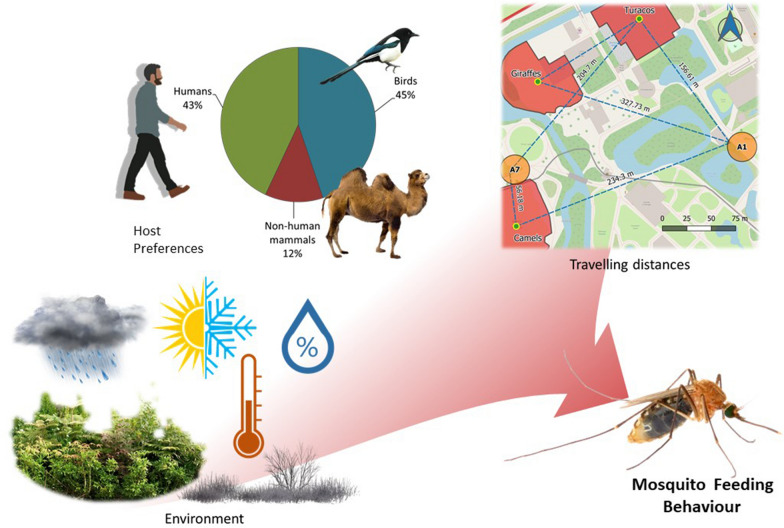

**Supplementary Information:**

The online version contains supplementary material available at 10.1186/s13071-021-04735-0.

## Background

The importance of mosquito-borne diseases continues to increase as invasive mosquito species and associated pathogens are detected in new habitats. The distribution expansion in southern Europe of the Asian tiger mosquito (*Aedes albopictus*), an effective vector of animal and human arboviruses, is just one example [1,3], and although this mosquito is not yet firmly established in the United Kingdom (UK), it could find extensive suitable habitats in the near future due to climate and environmental changes [4]. Blood-feeding behaviour and mosquito population density and longevity are key factors that influence the ability of mosquito populations to spread pathogens (a term known as vectorial capacity) [3, 5, 6]. Investigating vectorial capacity and its key factors is critical to understanding disease transmission risks by vectors and finding effective control approaches [5]. Nonetheless, studying mosquito blood-feeding behaviour is challenging, for three main reasons: (i) After feeding, mosquitoes seek a safe place for egg production; thus, they are not attracted to commonly used traps, which is reflected by their low proportion reported, even if the studies target them (310% [7,9]). (ii) Mosquitoes can disperse long distances when they look for potential hosts (e.g. *Aedes vexans* migrates to invade new habitats [10] and *Culex* spp. can travel for several kilometres [11]) and after blood-feeding (e.g. *Anopheles coluzzii* remains within 50m from its blood-host and disperses up to 250m after 60h [12]). (iii) Studying mosquito feeding patterns in natural settings is a problem of positive finding bias because engorged mosquitoes provide information about the hosts that they choose but not those that they avoid. Consequently, to thoroughly understand host preferences, techniques for capturing blood-fed mosquitoes should be improved and information about the abundance and density of potential hosts is needed, although in many settings this is not feasible to obtain. Knowing the identity and location of vertebrates in accessible environments, like zoological gardens, can therefore be advantageous in the study of mosquito host choices and post-feeding dispersal.

The zoo environment poses a risk of interspecific transmission of pathogens among vertebrate groups that usually cannot happen in natural environments [13]. In zoos, mosquitoes have access to a broad range of hosts and breeding sites due to the availability of endemic and exotic species of fauna and flora [13]. Mosquitoes collected at zoos have been found feeding on zoo vertebrates, free wild vertebrates, and humans, and mixed blood meals have been reported [8, 14, 15]. The relevance of mosquito ecology in zoos can be exemplified by the outbreak of West Nile virus (WNV), transmitted by *Culex* spp. mosquitoes, in the Bronx Zoo/Wildlife Conservation Park in relation to the first outbreak of WNV in America in humans [16]. Mosquito-borne diseases can be also a threat for the health of zoo vertebrates; avian malaria (caused by *Plasmodium* spp. haemosporidians) clearly exemplifies this, as it is a major cause of severe disease and death for captive penguins worldwide, and mosquito control and surveillance has been encouraged [17,19]. Lastly, exotic pathogens can be introduced and pose new risks for the health of humans and animals. For instance, Usutu virus (USUV) has caused mortality in wild birds (e.g. blackbirds, *Turdus merula*, and great grey owls, *Strix nebulosa*) [20] and has been detected in birds from zoos in central Europe [21]. Recently, it was detected in birds in the UK and, although the risk for humans is low to moderate, constant monitoring is recommended [22, 23]. Therefore, zoos are strategic sites for the surveillance of vector-borne pathogens due to their unique setting [13].

The vector potential of a mosquito depends on its physiological competence (if it can amplify the pathogen), the physiological competence of the host (if the pathogen can be amplified in the host), and the ecological capacity (if the environment supports transmission through enough mosquitohost contact). Mosquito species can exhibit diverse feeding patterns despite being exposed to the same vertebrates in a single location [24]. They can be generalists, feeding on a wide range of vertebrate species or groups, or specialists, preferring a narrow range of hosts [25, 26]. Generalist mosquitoes can facilitate pathogen transmission among unrelated species, and in such cases they are known as bridge vectors [1, 27,29]. However, the feeding of mosquitoes on hosts that cannot harbour the pathogen may lead to a dilution effect in pathogen transmission [14]. In the case of specialized mosquitoes, changes in the host community structure could force them to feed on unusual hosts [1], which could also facilitate interspecific transmission of pathogens. For example, *Aedes japonicus* that usually feeds on mammals (mammalophilic) has been found feeding on birds in some settings [30]. Likewise, when the mammalian-biting biotype of *Culex pipiens* (molestus) and its bird-feeding biotype (pipiens) hybridize, the resulting population may present mixed-host preferences and act as a bridge vector between birds and people [27]. Therefore, both generalist and specialist mosquitoes can function as bridge vectors, and the combination of vector host preference and host competence determine whether cross-species transmission occurs and whether a dilution or amplification effect is observed in relation to host diversity [31].

The aims of this study were to describe the environmental influence on feeding ecology, host choice patterns, and post-feeding dispersal of mosquitoes in two zoological gardens in the UK. This information improves our understanding of mosquito populations in zoos and provides insights into potential cross-species pathogen transmission risks. Over 2years, we identified blood meals from mosquitoes captured using traps for host-seeking mosquitoes and for gravid mosquitoes; techniques for capturing blood-engorged mosquitoes were implemented in a third sampling year in one zoological garden.

## Methods

### Sites and sampling methods

A general sampling of mosquitoes was performed for 2years (2017 and 2018) in Chester Zoo (Upton by Chester, Cheshire, UK) and for 1year (2017) in Flamingo Land (Kirby Misperton, North Yorkshire, UK). In 2019, we specifically sampled for blood-fed mosquitoes in Chester Zoo only.

The traps that we used in all samplings were the BG-Mosquitaire trap and the CDC-Gravid trap model 1712. The BG-Mosquitaire trap has a fan inside a plastic container that is connected to AC power; this trap captures host-seeking mosquitoes with its basic attractant, the BG-Sweetscent, based on lactic acid to mimic mammalian sweat [32]. The CDC-Gravid trap model 1712 consists of an electric fan powered by a 6V battery located inside a plastic cylinder and covered with a capture net, which is placed over a tray that contains 4L of infusion media as attractant for egg-laying mosquitoes [33]. The infusion media is prepared with tap water (40L), hay (200g), brewers yeast (2g), and milk powder (2g), and the mix is stored for at least 1week before use [34].

**Fig. 1:**
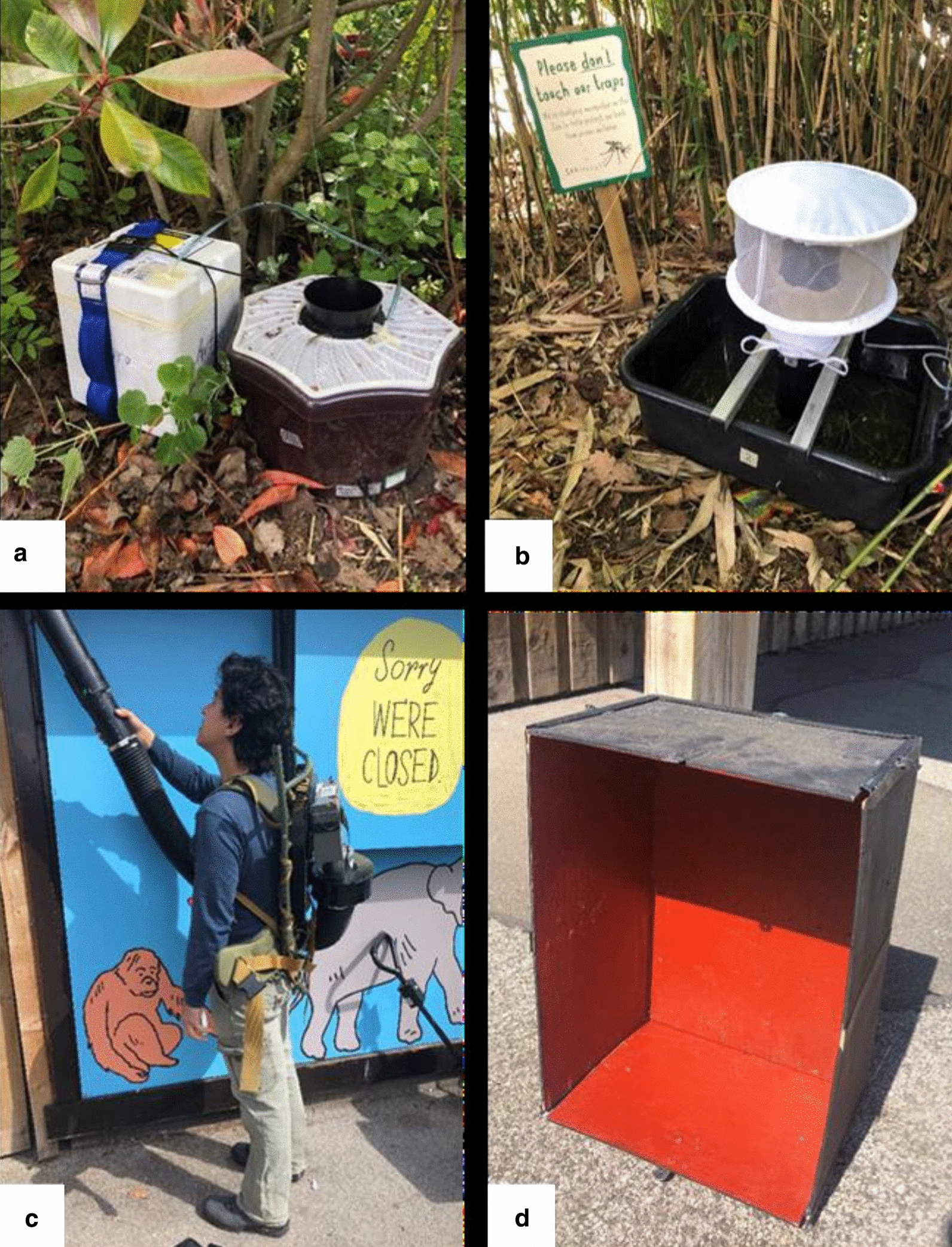
Trapping methods used for mosquito sampling. **a** BG-Mosquitaire trap (upgraded with dry ice for the 2019 season); **b** CDC-Gravid trap model 1712; **c** Improved CDC-Backpack Aspirator model 1412 (only used in 2019); **d** resting box trap (only used in 2019). Pictures by FT

For the 2019 season, four capture methods were used: BG-Mosquitaire trap upgraded with dry ice as source of CO_2_, CDC-Gravid trap, resting trap, and aspiration in mosquito resting areas. The BG-Mosquitaire traps were operated using the basic attractant (BG-Sweetscent) and dry ice to increase the chances of capturing host-seeking mosquitoes and the species range [35]. For this, a polyurethane box was placed next to each trap, connected to the trap with a plastic tube, and sealed with silicone glue; the lid was sealed to the box with tape and a strap with a combination lock to prevent accidental opening. The CDC-Gravid traps were used as described previously. The resting trap is a 403030cm wooden box with an open side. There is no attractant used; mosquitoes simply go inside the box to rest, especially during daytime. We placed these traps with the open face on the underside, leaning against a structure (e.g., walls, trees, or rocks) at 45 to allow a gap for mosquito entry. We used an Improved CDC-Backpack Aspirator model 1412 [36] to aspirate the inside of the resting traps. Finally, the same aspirator was used to systematically aspirate potential resting places, such as vegetation, walls, fences, and the outside of buildings, for 5 min in each sampling area (Fig.1).

Sampling areas were defined as 30m-diameter circles that contained one trap of each type, and set in public areas, staff areas, or vertebrate exhibits. Inside sampling areas, the traps were at least 10m apart from each other to minimize interference between them, and located considering safety for people and animals, logistic implications, surrounding vegetation (avoiding coverage at least 2m above them to reduce the possibility of leaves interfering with the traps fan), and with protection from direct sunlight and rain when possible.

### Sampling protocols

**Fig. 2:**
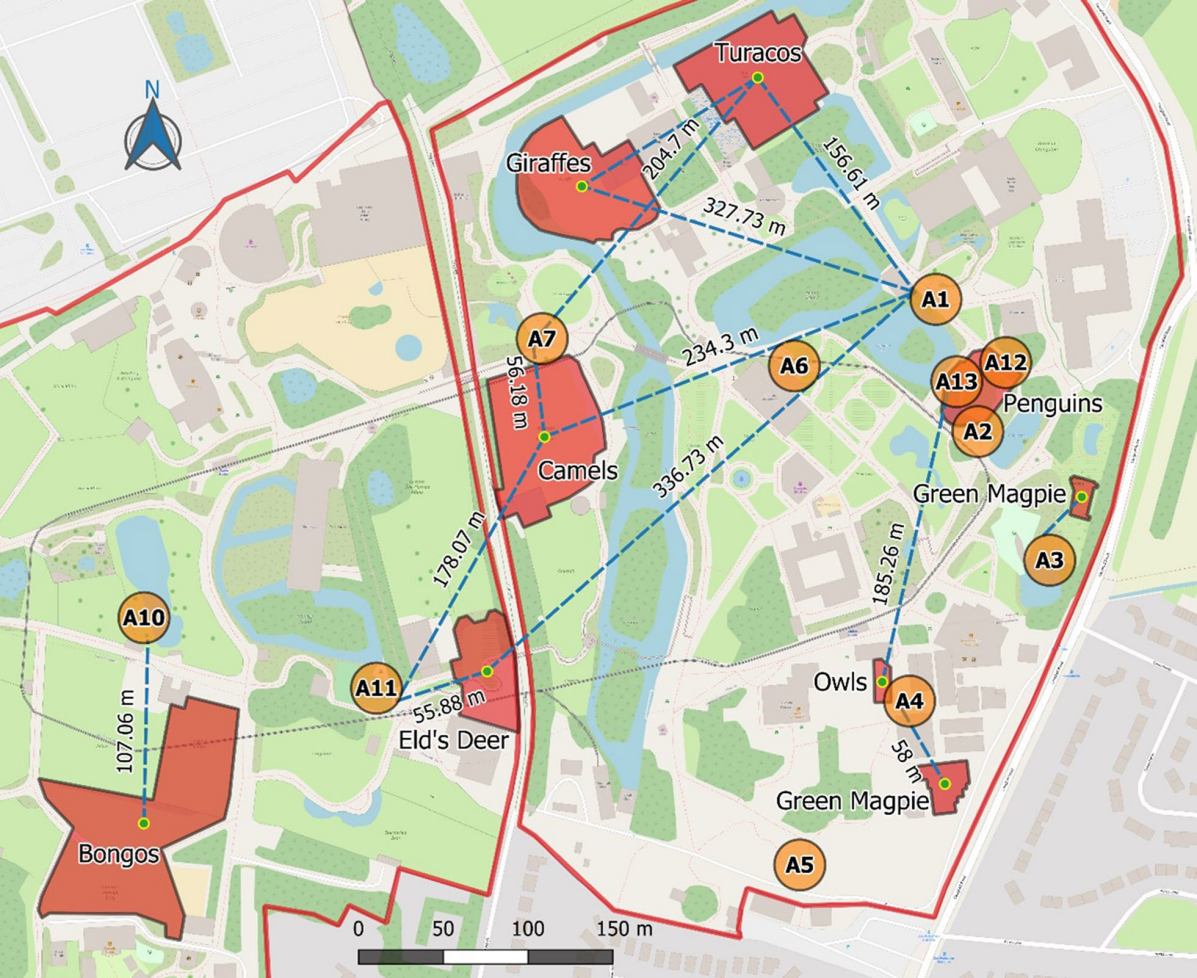
Sampling areas and minimum travelling distances of blood-fed mosquitoes in Chester Zoo. Not all distances are shown. Red areas: zoo vertebrate exhibits; orange circles: sampling areas; green dots: exhibit centroids; dotted lines: minimum travelling distances; red line: zoos perimeter. Area A13 was not active in the 2017 sampling; areas A5, A6, and A7 were not active in the 2018 sampling; areas A4, A5, A6, A7, A10, and A11 were not active in the 2019 sampling

**Fig. 3:**
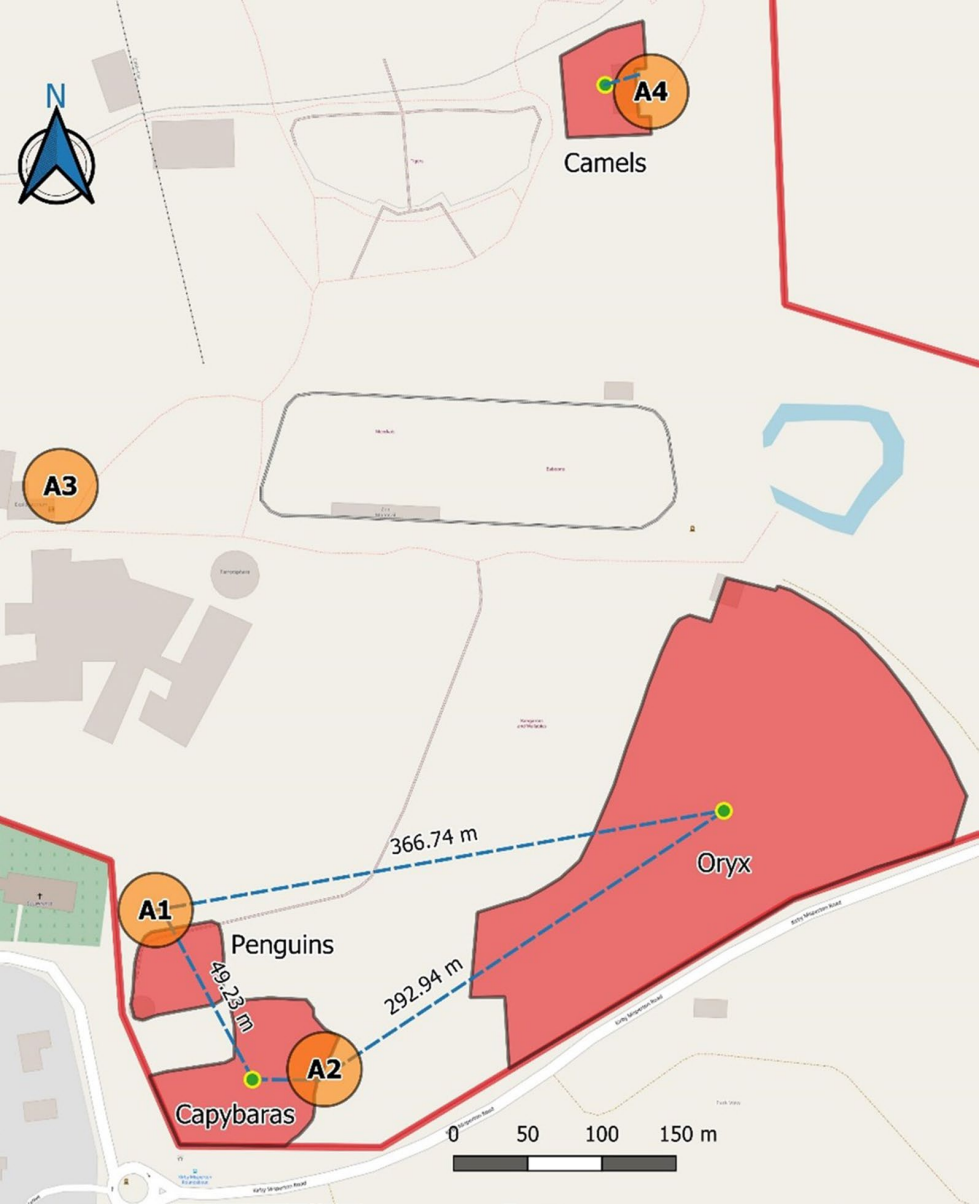
Sampling areas and minimum travelling distances of blood-fed mosquitoes in Flamingo Land, 2017. Not all distances are shown. Red areas: zoo vertebrate exhibits; orange circles: sampling areas; green dots: exhibit centroids; dotted lines: minimum travelling distances; red line: zoos perimeter

We established ten sampling areas in Chester Zoo in the 2017 season; in 2018 we discontinued three areas due to low catches, and added a new area located inside the penguin exhibit (Fig.2). Sampling took place for 32weeks in Chester Zoo in 2017 (May to December) and for 31weeks in 2018 (April to November). In Flamingo Land, we established four sampling areas (Fig.3), which were sampled for 25weeks in 2017 (June to November). In both sites, BG-Mosquitaire traps were emptied twice a week, once after six consecutive nights and once after one night; the CDC-Gravid traps were operated one night per week. This resulted in a 6-day collection from the BG-Mosquitaire traps followed by a 1-day collection from both traps every week.

In addition, specific sampling of blood-fed mosquitoes took place in Chester Zoo for 11weeks (April to July) in 2019 using five sampling areas close to the penguin exhibit (Fig.2). In this occasion, the weekly schedule consisted of one collection from the BG-Mosquitaire traps after 5days with the basic attractant plus two collections after 1day each adding dry ice, two aspirations from resting traps and two from surrounding areas, and one collection from the CDC-Gravid traps after 2days of operation.

### Environmental monitoring

The environment was monitored for landscape and weather variables. Landscape variables were categorized with three possible values and included vegetation (dense, medium, or scarce), proximity to suitable mosquito oviposition sites (close, medium, or remote), mosquito resting areas (abundant, medium, or rare), and proximity to zoo vertebrate exhibits (close, medium, or remote). The vegetation value was changed to scarce for all traps when foliage decreased in autumn. The definition of variable values can be found in Additional file 1: Table S1 and the corresponding values per trap in Additional file 1: Table S2.

Weather variables included regional temperature, relative humidity, wind speed, and precipitation. Daily values were obtained using the R package Climate, which extracts information from the OGIMET Weather Information Service (www.ogimet.com). We downloaded data from the closest inland weather stations; for Chester Zoo it is Hawarden (13km) and for Flamingo Land, Linton On Ouse (52.2km). We did not use the data from stations closer to Flamingo Land because they are on the coast and, therefore, influenced by marine climate.

In 2017 and 2018 we used Tinytag loggers, 13 of the Plus 2 TGP-4500 model and two of the Ultra 2 TGU-4500 model (Gemini Data Loggers, UK), to record local temperature and humidity. Loggers were placed next to the BG-Mosquitaire traps, programmed to record every hour, and average readings were used. The loggers were not used in 2019.

### Laboratory protocols

Collected nets were transported in a cooled icebox and placed into a 20C freezer on arrival for approximately 2h. Mosquitoes were separated from accompanying insects and identified by morphology under a stereomicroscope following identification keys [10, 37]. During the morphological identification, the abdomens of the blood-fed mosquitoes were cut off using entomological forceps and disposable scalpels; the materials were cleaned with 70% ethanol and DNA Away between specimens. The abdomens were stored in individual reaction tubes at 80C for no more than 3weeks until processing. As field-captured mosquitoes have different degrees of blood digestion that affects host identification [38], mosquitoes were selected for analysis if they were engorged with a red, dark red, or blackish abdomen indicating blood content. This is equivalent to the stages II and III of the Sella classification system [9, 39].

Mosquito abdomens were homogenized with 200l of phosphate-buffered saline (PBS) per sample [7] using sterile plastic pestles or with a stainless-steel bead and a QIAGEN TissueLyser at frequency of 24Hz per second for 2min. For DNA extraction, we used the OMEGA Bio-Tek E.Z.N.A Tissue DNA Kit following the manufacturers instructions; extracts were stored at 4C until further processing for no more than 2weeks and at 20C for long-term storage. Afterwards, the polymerase chain reaction (PCR) protocol and primers proposed by Alcaide et al. [40] were used to obtain a 758-base pair amplicon of the cytochrome *c* oxidase subunit I (COI) gene. Negative controls (nuclease-free water) were added every five samples, and DNA extract from liver of black-headed gull (*Larus ridibundus*) was used as a positive control. The amplification was verified by electrophoresis in a 1% agarose gel. Positive PCR products were sent to Macrogen Europe B.V. for Sanger sequencing using the M13 primer in the forward direction. All the PCR-negative samples were tested at least twice.

Successful sequences were edited and analysed using BioEdit software and compared to the reported sequences in the Basic Local Alignment Search Tool (BLAST), optimized for the highly similar sequences, and the Barcode of Life Data System (BOLD). The most similar sequences were aligned and compared using BioEdit to find the best match considering the identity and query covers and excluding wild native species absent in the area and exotic species not included in the zoo collections. With the same software, we inspected the electropherograms for double peaks in a single base position, which, if consistent, indicate that the blood meal was mixed, with the mosquito having fed on more than one host species [40].

Mosquitoes belonging to the *Culex* spp. genus include, in the UK, two sympatric species that are indistinguishable morphologically, *Cx*. *pipiens* and *Cx. torrentium*. Females from this genus were identified using the PCR and enzyme restriction protocol developed by Hesson et al. [41]. Likewise, *Cx. pipiens* has two morphologically identical biotypes with different biological traits and epidemiologic roles, pipiens and molestus, the first of which is reported predominately as ornithophilic and the second as mammalophilic [27, 42, 43]. We found *Cx. pipiens* feeding on humans, so we tested them using the multiplex PCR protocol proposed by Bahnck et al. [44] to differentiate their biotype.

### Flying distances

Knowing the zoo vertebrates on which mosquitoes have fed, we estimated the distance between the relevant vertebrate exhibits and the location of the trap where the blood-fed mosquitoes were captured. Species360 Zoological Information Management Software (ZIMS) [45] was used to determine the location of the zoo vertebrates at the time when mosquitoes were captured. The Open Street Map layer was used in QGIS 3.2 software to delineate the polygons of the exhibits. Then, the centroid of the exhibits was estimated, and the minimum travelling distance of mosquitoes was represented as the length of the line from the centroid to the corresponding trap.

### Data analysis

Blood-fed mosquito proportions were compared separately by zoo and sampling year to detect differences in distribution (by sampling area), seasonality (monthly), and capture method, and host choices were compared considering all vertebrate group (birds, non-human mammals, and humans) and mosquito species. A chi-square test of independence was used exploring the test residuals to find sources of significance or Fishers exact test of independence was chosen when appropriate. Additionally, log odds ratios were estimated to compare mosquito feeding patterns for host groups in pairs by sampling; for this, 0.5 was added to all values to allow the calculation when a mosquito did not feed in one host group. To assess differences in travelling distances by mosquito species, a KruskalWallis test was used. Specimens with damaged or missing abdomens were excluded and only completely identified mosquitoes were included in the analyses by species.

To understand environmental influences in blood-fed mosquito captures, we used generalized linear models (GLMs) for a proportion response using a binomial family (or quasibinomial in case of overdispersion) and a logit link, with a backwards elimination process to find minimal adequate models. Mosquito data were consolidated per week, and weather data were averaged for the week before collection. Models were constructed for each sampling separately including weather and landscape variables, and for the overall mosquito collection using weather variables. Regional and local temperature data were expected to be strongly correlated, so the one producing the best model fit was used. All tests were done with the R software, and for the models the MASS package was used [46].

## Results

Excluding males and mosquitoes with damaged abdomens, the number of blood-fed mosquitoes captured (and percentage of all female mosquitoes caught) in Chester Zoo was 213 (3.5%) in 2017, 245 (9.7%) in 2018, and 107 (4.1%) in 2019. We caught 75 (7.2%) in Flamingo Land in 2017. In total, we caught 640 blood-fed mosquitoes (5.2%) (Table 1). Most blood-fed mosquitoes in both sites were *Culex pipiens* (*n*=497) and *Culiseta annulata* (*n*=81), although we also captured one *Anopheles claviger* and four *An. maculipennis* s.l. in 2018 and three *An. maculipennis* s.l. in 2019. Fifty-five mosquitoes were damaged and unable to be identified to species level. PCR testing confirmed that all engorged *Culex* spp. mosquitoes were *Cx. pipiens* and all of those which fed on humans were *Cx. pipiens* biotype pipiens. Other species captured included *Aedes annulipes*, *Ae. vexans*, *Ae. detritus*, *An. plumbeus*, *An. claviger*, *Cs. morsitans*, and *Cx. torrentium*, although these mosquitoes were not blood-fed and were captured in low numbers.

**Table 1:** Blood-fed mosquitoes captured and testing results per sampling

Sampling	Blood-feds/total captured^a^ (%)					PCR positives (%)^b^	Sequencing matches (%)^c^	Identified hosts (%)^d^				
	BG-Mosquitaire	CDC-Gravid	Resting Trap	Aspiration	Total			Birds	Non-human mammals	Humans	Total	Mixed hosts
Chester Zoo 2017	132/3424 (3.9)	81/2593 (3.1)			213/6017 (3.5)	95 (44.6)	75 (78.9)	31 (38.8)	9 (11.3)	40 (50.0)	80 (100)	5 (6.6)
Chester Zoo 2018	185/1988 (9.3)	60/545 (11)			245/2533 (9.7)	56 (22.9)	45 (80.4)	24 (52.2)	5 (10.9)	17 (37.0)	46 (100)	1 (2.2)
Chester Zoo 2019	39/1505 (2.6)^e^	43/971 (4.4)	12/65 (18.5)	13/59 (22)	107/2600 (4.1)	23 (21.5)	20 (86.9)	18 (90.0)	0 (0)	2 (10.0)	20 (100)	0 (0)
Flamingo Land 2017	46/862 (5.3)	29/186 (15.6)			75/1048 (7.2)	30 (40)	19 (63.3)	1 (5.3)	6 (31.6)	12 (63.2)	19 (100)	0 (0)
Total	402/7779 (5.2)	213/4295 (4.9)	12/65 (18.5)	13/59 (22)	640/12,198 (5.2)	204 (31.9)	159 (77.9)	74 (44.8)	20 (12.1)	71 (43.0)	165 (100)	6 (3.6)

^a^Excluding males and mosquitoes with damaged abdomens

^b^Over blood-fed mosquitoes

^c^Over PCR positives

^d^Mixed blood meals were considered as a choice for both hosts

^e^These traps were upgraded with CO_2_ for this sampling

### Methods assessment

Proportions of blood-fed mosquitoes were compared by traps excluding females with abdominal damage and using only the 1-day collections in the case of the BG-Mosquitaire traps. There were no significant differences either in Chester Zoo in 2017 (*X*^2^=0.047, df=1, *P*=0.829) or 2018 (*X*^2^=1.373, df=1, *P*=0.241), or Flamingo Land (*X*^2^=2.688, df=1, *P*=0.101). Nonetheless, we observed a significant difference comparing all methods used in 2019 (Fishers exact test, *P*<0.001): a higher proportion of blood-fed mosquitoes were captured in resting traps and by aspiration than by BG-Mosquitaire traps or CDC-gravid traps. The success of PCR amplification per sampling varied from 21.5 to 44.6% and was 31.9% overall. From these samples, the sequencing success varied from 63.3 to 86.9%, being 77.9% overall (Table 1).

### Host patterns

The vertebrate hosts were identified from 159 blood-fed mosquitoes and were grouped as birds (*n*=74), non-human mammals (*n*=20), and humans (*n*=71). Two different hosts were identified from the same mosquito in six occasions which were counted as a choice for both hosts (Table 1). The most common species of free wild birds were passerine residents, Eurasian jackdaw (*Corvus monedula*) (*n*=11), Eurasian magpie (*Pica pica*) (*n*=9), and Eurasian blackbird (*Turdus merula*) (*n*=8), although Mallard (*Anas platyrhynchos*), another resident (order Anseriformes), was also common (*n*=7). From the five species of zoo birds identified, the Schalows turaco (*Tauraco schalowi*) (*n*=5), Humboldt penguin (*Spheniscus humboldti*) (*n*=4), and Javan green magpie (*Cissa thalassina*) (*n*=2) were more frequently identified. The zoo non-human mammals most often found were the Bactrian camel (*Camelus bactrianus*) (*n*=6), Elds deer (*Rucervus eldii thamin*) (*n*=3), scimitar-horned oryx (*Oryx dammah*) (*n*=2), and capybara (*Hydrochoerus hydrochaeris*) (*n*=2).

**Fig. 4:**
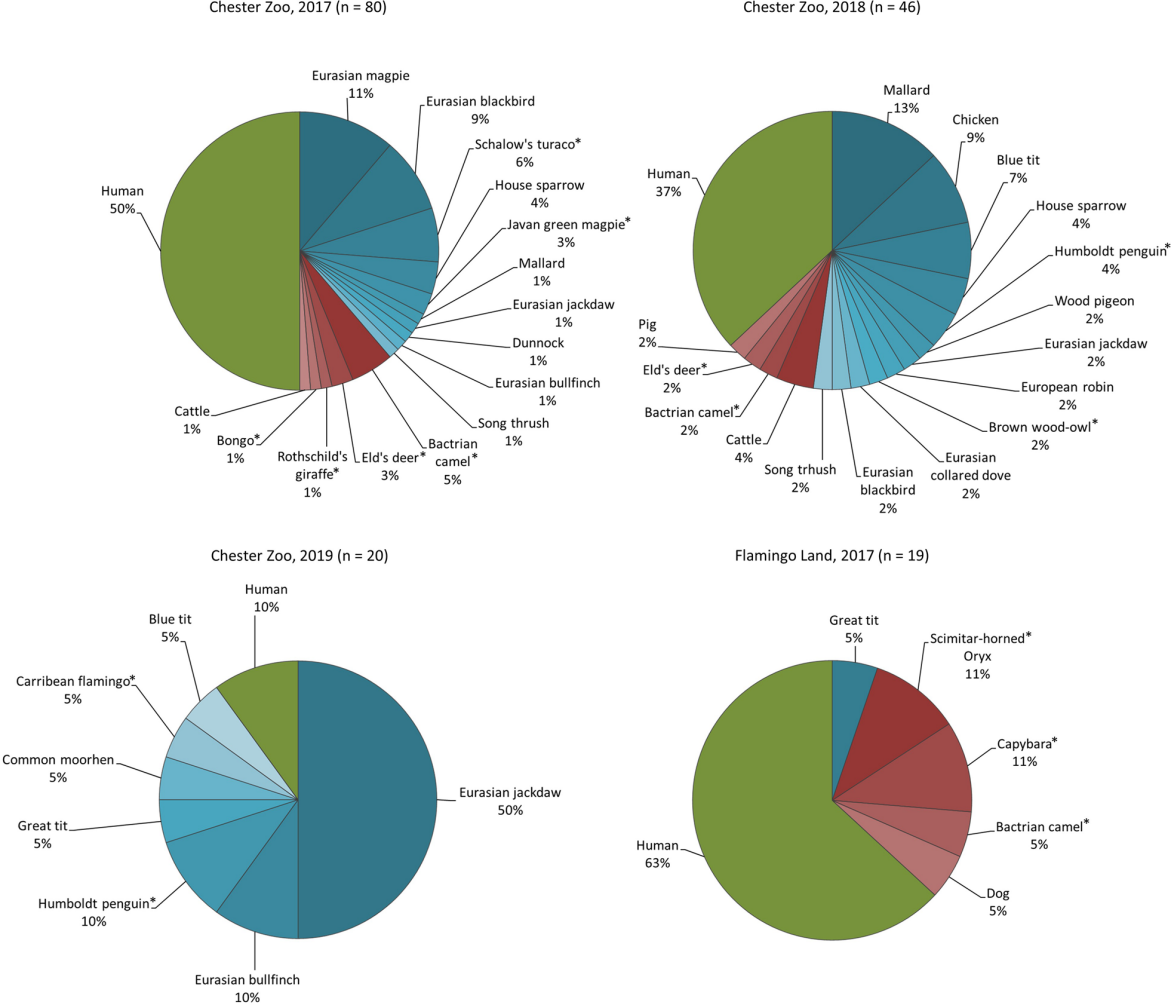
Host patterns of blood-fed mosquitoes. Birds in shades of blue, non-human mammals in shades of red. Zoo vertebrates are indicated with an asterisk

**Fig. 5:**
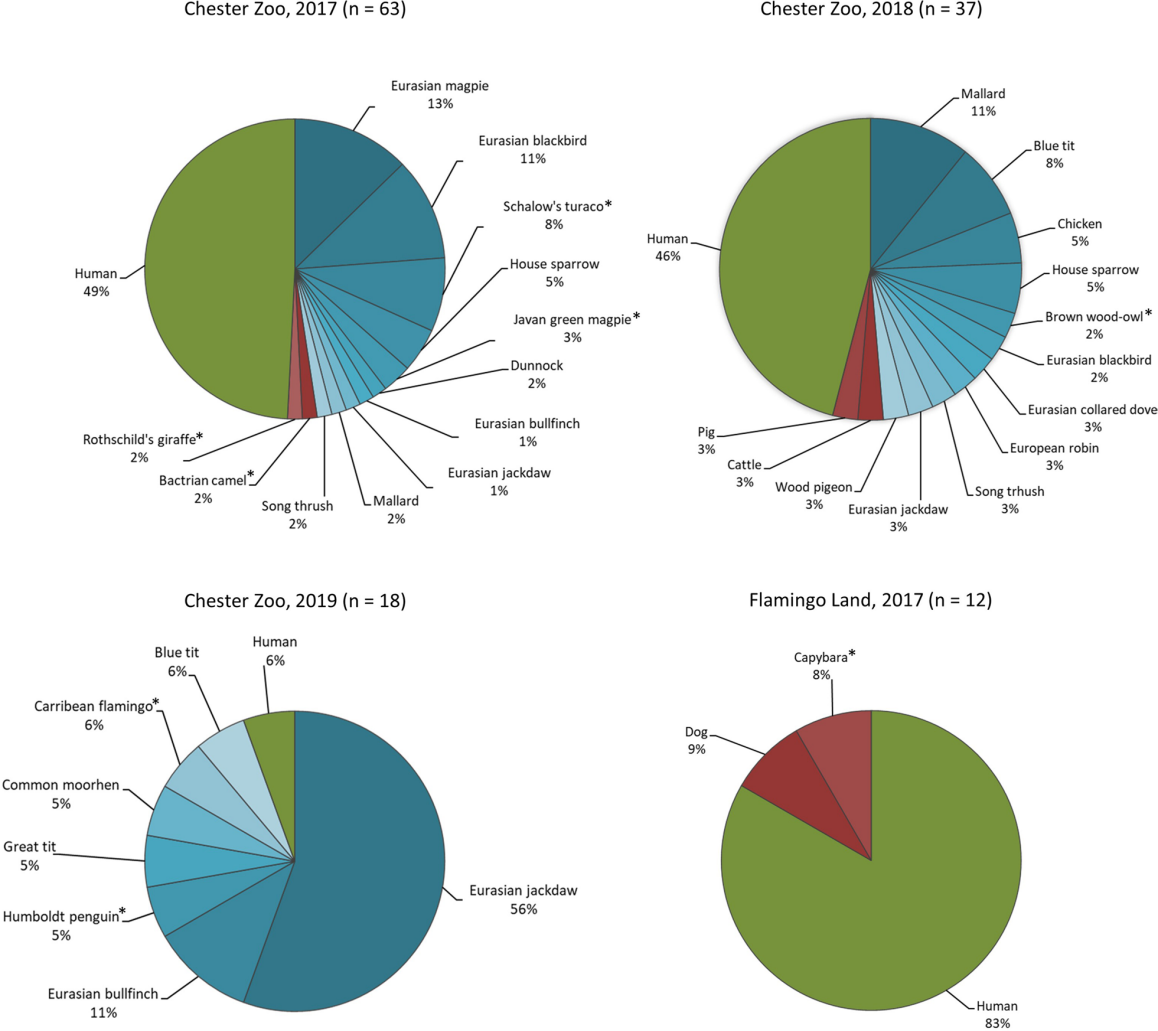
Host patterns of *Culex pipiens*. Birds in shades of blue, non-human mammals in shades of red. Zoo vertebrates are indicated with an asterisk

In Chester Zoo, eight species of free wild birds, two zoo birds, four zoo mammals, one cattle, and 37 samples from human were identified in 2017 (Additional file 1: Table S3). The following year, we found nine species of free wild birds, two zoo birds, one chicken, one cattle, one pig, two zoo mammals, and 17 humans (Additional file 1: Table S4). In 2019, we identified five species of free wild birds, two zoo birds, and two humans (Additional file 1: Table S5). In Flamingo Land (2017), one free wild bird, three species of zoo mammals, one dog, and 10 humans were identified (Additional file 1: Table S6). Proportions of hosts are shown overall per sampling in Fig.4, for *Cx. pipiens* in Fig.5, and for the other species in Additional file 1: Fig. S1.

**Fig. 6:**
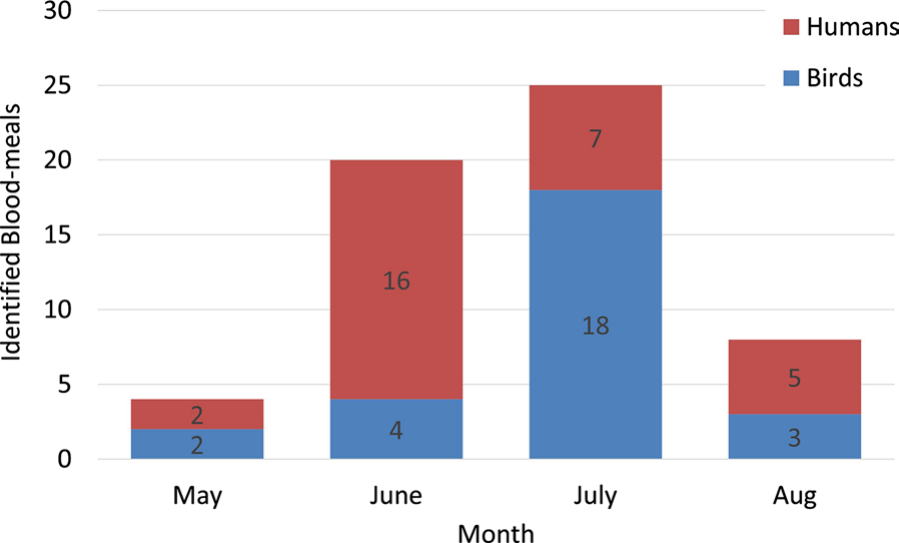
Host patterns of *Culex pipiens* during 2017 in Chester Zoo

Significant differences were found in Chester Zoo in 2017 (Fishers exact test *P*<0.001) where *Cx. pipiens* preferred to feed on birds and humans, and *Cs. annulata* preferred to feed on non-human mammals and humans. In the following year, *Cx. pipiens* also preferred birds and humans and *Cs. annulata* preferred non-human mammals (Fishers exact test *P*=0.025). In 2019, the number of samples per group was not sufficient for statistical analysis, as most of the *Cx. pipiens* were feeding on birds (*n*=17), but a choice of *Cx. pipiens* for birds and of *An. maculipennis* s.l. for humans was observed. The difference in host patterns was also significant in Flamingo Land (Fishers exact test *P*=0.021), with *Cx. pipiens* preferring humans and *Cs. annulata* non-human mammals. Number and proportion of host choices per mosquito species and sampling are presented in Table 1, and log odds ratios for host choices of mosquitoes in Table 2. Analysing differences in host patterns by month, we found a significant difference in Chester Zoo in 2017 (Fishers exact test *P*=0.003); *Cx. pipiens* preferred to feed on humans during June and on birds during July (Fig.6). Data were insufficient for other comparisons, or differences were not significant.

**Table 2:**
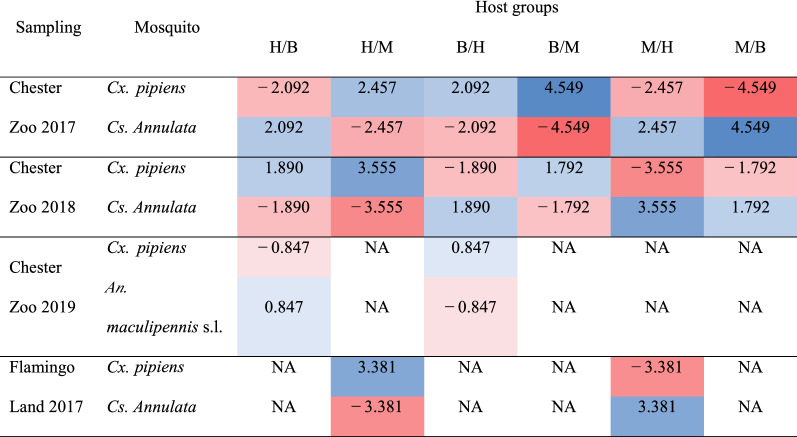
Log odds ratios for mosquito host choices

The intensity of the shade indicates the strength of association, positive (blue) or negative (red)

*H* humans, *B* birds, *M* non-human mammals, *NA* not applicable due to none of the mosquito species feeding on one of the host groups

### Distribution of feeding activity

**Fig. 7:**
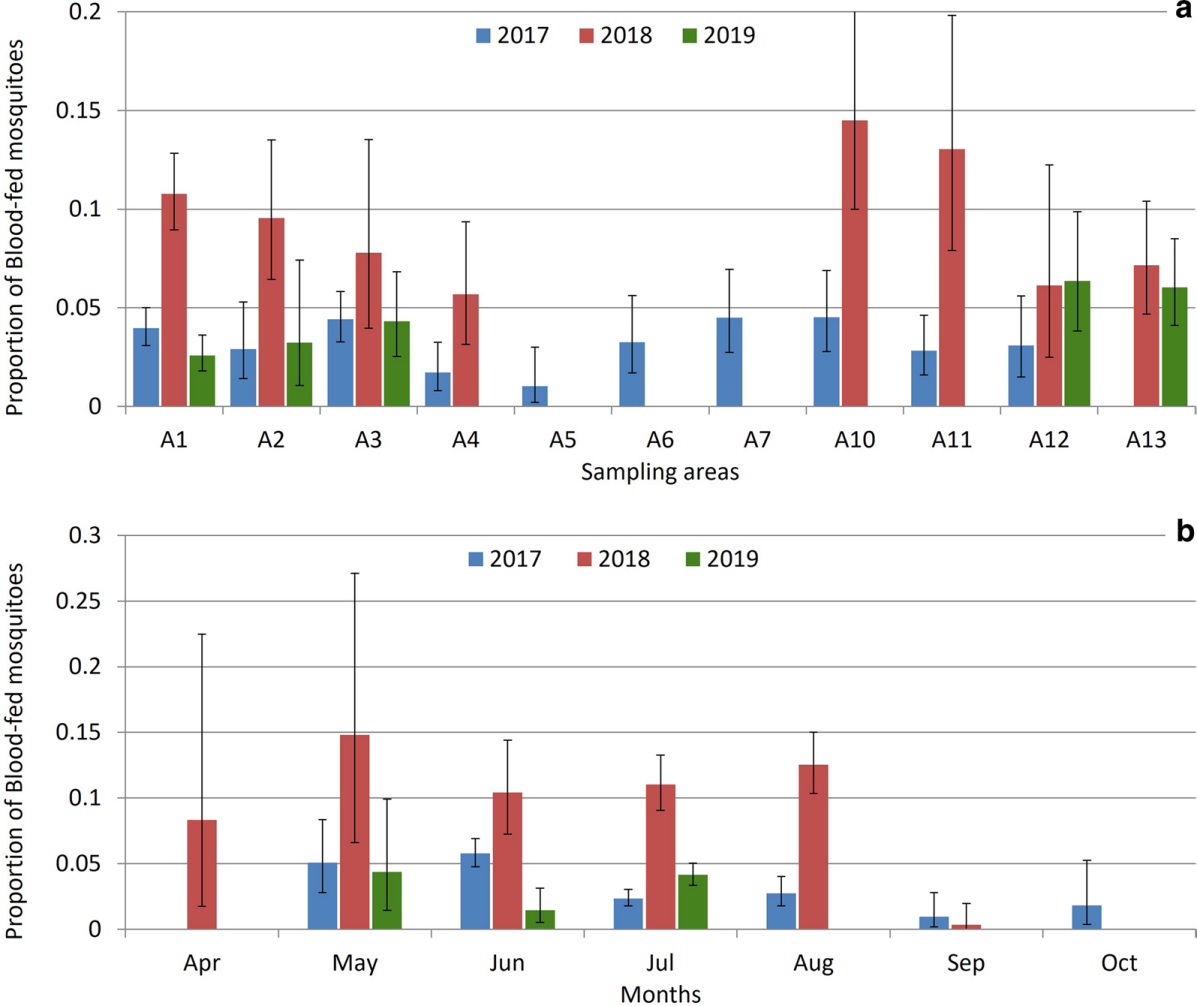
Proportion of blood-fed mosquitoes over the total mosquito catch by sampling areas (**a**) and months (**b**) in Chester Zoo. Error bars: 95% confidence intervals. Sampling areas and months without bars were not sampled in that year. Sampling areas can be consulted in Fig.2

**Fig. 8:**
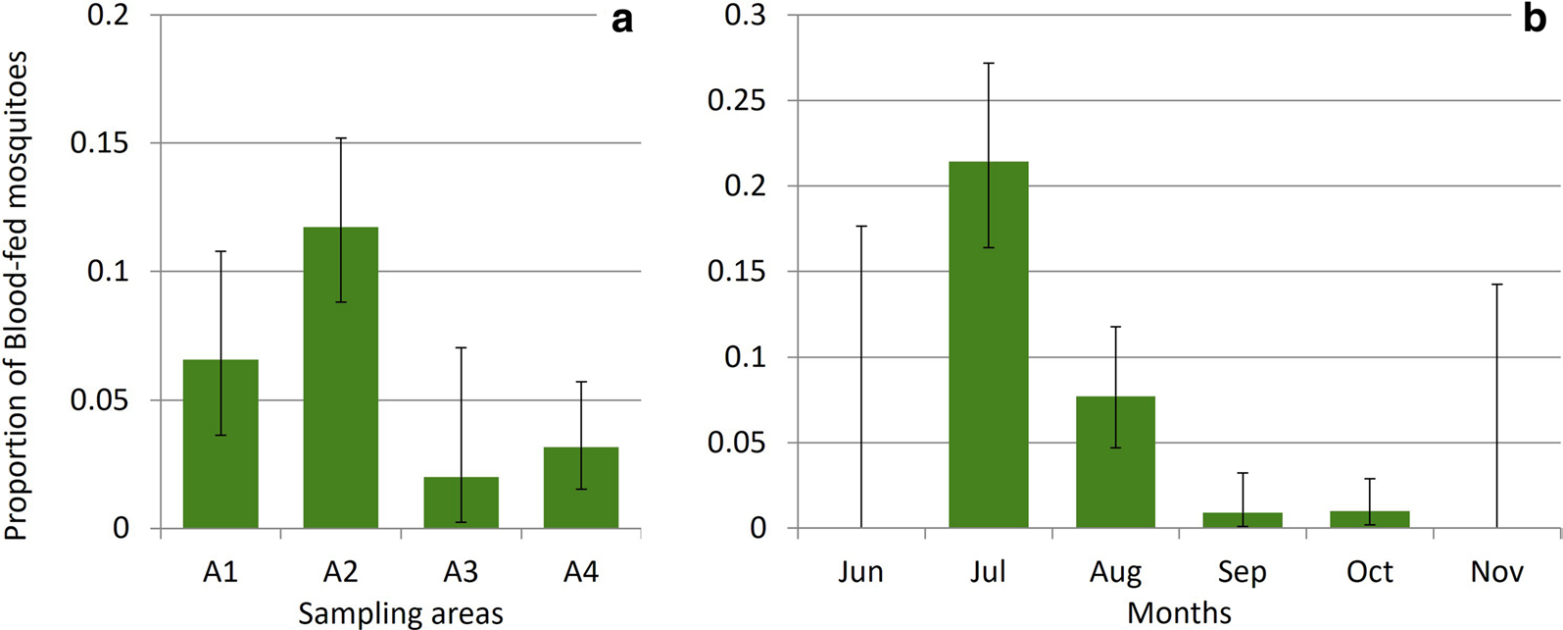
Proportion of blood-fed mosquitoes over the total mosquito catch by sampling areas (**a**) and months (**b**) in Flamingo Land, 2017. Error bars: 95% confidence intervals. Sampling areas are presented in Fig.3

We observed significant differences in blood-fed mosquito catches among sampling areas in all our samplings. In Chester Zoo, during the first year, areas A1 and A3 captured the largest numbers of mosquitoes and highest proportions of blood-feds; areas A7 and A10 also captured more blood-fed mosquitoes than expected randomly but not in relation to a large mosquito catch (*X*^2^=17.556, df=9, *P*=0.041). The following year, area A10 captured more blood-fed mosquitoes, along with areas A1 and A11 (*X*^2^=17.894, df=7, *P*=0.013). In the last year, areas A12, A,13 and A3 yielded high proportions of blood-fed mosquitoes, and in this occasion, captures in area A1 were less than expected by chance (*X*^2^=16.55, df=4, *P*=0.002) (Fig.7a). We also found a significant difference in Flamingo Land related to a large catch of blood-fed mosquitoes in area A2 and smaller catches in areas A3 and A4 (*X*^2^=24.868, df=3, *P*<0.001) (Fig.8a).

### Seasonality of feeding activity

The proportion of blood-fed mosquitoes trapped also changed among months. A significantly higher proportion of blood-fed mosquitoes in June and May and a lower proportion in July were observed in Chester Zoo in the first year of sampling (*X*^2^=50.596, df=6, *P*<0.001). In 2018, catches larger than expected randomly were observed in July and August and smaller than expected in September and October (*X*^2^=54.346, df=6, *P*<0.001). The sampling in 2019 captured more blood-fed mosquitoes in July and May and less in June (*X*^2^=7.15, df=2, *P*=0.028) (Fig.7b). Likewise, in Flamingo Land a higher proportion of blood-fed mosquitoes were observed in July and August compared to other months (*X*^2^=106.51, df=5, *P*<0.001) (Fig.8b).

### Travelling distances

We identified 14 zoo host species from mosquito blood meals in Chester Zoo in 2017, five in 2018, three in 2019, and five in Flamingo Land. The minimum travelling distance observed overall was 13.72m and the maximum, 366.74m (Table 3). By species, the average minimum travelling distance was 122.5m for *Cx. pipiens* (median=113.3m), 131.8m for *Cs. annulata* (median=56.2m), and 20.3m for *An. maculipennis* s.l. (median=20.3m) (Figs. 1, 2). A comparison by species (excluding *An. maculipennis* s.l. due to low numbers) showed no significant difference overall (KruskalWallis, *X*^2^=0.279, df=1, *P*=0.597).

**Table 3:** Minimum travelling distances of blood-fed mosquitoes that fed on zoo vertebrates

Sampling season	Mosquito	Zoo vertebrate		Sampling area	Minimum flying distance (m)
		Scientific name	Common name		
Chester Zoo 2017	*Cx. pipiens*	*Camelus bactrianus*	Bactrian camel	A1	234.3
		*Cissa thalassina*	Javan green magpie	A4	58
		*Cissa thalassina*	Javan green magpie	A3	34.16
		*Giraffa camelopardalis rothschildi*/*Tauraco schalowi*	Rothschilds giraffe/Schalows turaco	A1	303.28^a^
		*Tauraco schalowi*	Schalows turaco	A1	168.58
		*Tauraco schalowi*	Schalows turaco	A1	168.58
		*Tauraco schalowi*	Schalows turaco	A7	204.7
	*Culiseta* spp.	*Camelus bactrianus*	Bactrian camel	A7	56.18
		*Camelus bactrianus*	Bactrian camel	A7	56.18
		*Camelus bactrianus*	Bactrian camel	A7	56.18
		*Rucervus eldii thamin*	Elds deer	A1	336.73
		*Rucervus eldii thamin*	Elds deer	A11	55.88
		*Tragelaphus eurycerus*	Bongo	A10	107.06
	Unknown^b^	*Tauraco schalowi*	Schalows turaco	A1	156.61
Chester Zoo 2018	*An. maculipennis*	*Spheniscus humboldti*	Humboldt penguin	A2	21.34
	*Cx. pipiens*	*Strix leptogrammica*	Brown wood Owl	A13	185.26
	*Culiseta* spp.	*Camelus bactrianus*	Bactrian camel	A11	178.07
		*Rucervus eldii thamin*	Elds deer	A11	55.88
	Unknown^b^	*Spheniscus humboldti*	Humboldt penguin	A2	21.34
Chester Zoo 2019	*Cx. pipiens*	*Phoenicopterus ruber*	Caribbean flamingo	A1	28.41
	*Cx. pipiens*	*Spheniscus humboldti*	Humboldt penguin	A12	13.72
	*An. maculipennis*	*Spheniscus humboldti*	Humboldt penguin	A12	19.19
Flamingo Land	*Cx. pipiens*	*Hydrochoerus hydrochaeris*	Capybara	A2	46.67
	*Culiseta* spp.	*Camelus bactrianus*	Bactrian camel	A4	24.76
		*Hydrochoerus hydrochaeris*	Capybara	A1	49.23
		*Oryx dammah*	Scimitar-horned oryx	A1	366.74
	Unknown^b^	*Oryx dammah*	Scimitar-horned oryx	A2	292.94

^a^Mixed blood meals, includes the distance between the exhibits of both vertebrates and the trap

^b^These mosquitoes were only identified to the Culicinae subfamily due to damaged legs. Sampling areas are presented in Fig.2 for Chester Zoo and Fig.3 for Flamingo Land

### Environmental factors

Six variables showed a significant influence on blood-fed mosquito captures, although the direction of influence was not consistent. Temperature had a positive influence in blood-fed mosquito captures in four models (close to significance in one, *P*=0.073) and a negative influence in another, humidity had a negative influence in three models and a positive influence in one, precipitation was a positive influence in three models, wind speed was positively influencing in one model and negatively in another, and scarce vegetation and close distance to zoo vertebrate exhibits had a positive influence in one model. A summary of estimates and *P* values can be found in Additional file 1: Table S7.

## Discussion

As blood-engorged mosquitoes tend to be captured in low proportions in conventional mosquito traps (e.g. [35]), it is not clear whether blood-fed mosquitoes were attracted by the lactic acid of host-seeking mosquito traps because they were looking for a second blood meal, because of the CO_2_ of fermenting media of gravid traps, or because they prefer to rest near potential oviposition sites. Alternatively, they may be attracted by the dark colour and location of the traps, perceived as potential resting sites. However, we cannot exclude the possibility that they were randomly captured, as no significant differences were found between BG-Mosquitaire and CDC-Gravid trap types. We captured higher proportions of blood-fed mosquitoes using resting traps and aspirating resting areas, as reported by other authors [7, 14, 47]; therefore, these techniques are recommended for blood-engorged mosquito sampling. In some cases, the use of resting traps has failed [14, 48], and thus their positioning and orientation could be a determinant and should ensure that mosquitoes get protection from direct sunlight, rain, and wind.

Martinez-de la Puente et al. [1] and Reeves et al. [38], using the same PCR protocol for blood-meal identification as we used, showed that storage time and post-feeding time have a significant and negative effect on amplification success. Other authors had higher amplification success rates than ours (around 7080%) using the same protocol, but in these cases, they collected mosquitoes after 24-hour periods [49, 50]. However, Abella-Medrano et al. [1], using the same protocol and collecting the mosquitoes shortly after feeding, identified only 11% of the blood-fed mosquitoes; in this case, the authors attributed interference to the advanced stage of blood digestion [14]. Authors using other PCR protocols reported similar amplification success rates (around 7080%) [21, 32] although Goodman et al. [11] mentioned lower success with *Culex* mosquitoes (31.7%) in relation to sample preservation [21]. Our blood-meal identification success could have been influenced by the time before collecting the mosquitoes and weather conditions. If the mosquitoes remained alive, they continued digesting the blood, and if dead, they started to desiccate. We observed more mosquitoes completely dehydrated, despite their evident blood-fed status, during the 2018 sampling in Chester Zoo, which was drier and hotter than the other sampling years. Abundant mosquitoes were collected in some samplings requiring more time at this step, which could also have affected the number of successful amplifications. To improve the results, storing the samples at 80C or in filter paper and processing them promptly, prioritising engorged females, has been recommended [7, 9, 38]; this also minimises physical damage, facilitating mosquito morphological identification.

We confirmed the intrinsic feeding pattern of *Culex pipiens* for birds, mainly free wild birds and some birds from the zoos collections, and of *Culiseta annulata* for mammals. *Cx. pipiens* is primarily an ornithophilic species [42, 51]; however, we observed high proportions of human hosts in all our samplings, except for 2019. Heym et al. [8] and Brstler et al. [52] similarly found mosquitoes feeding on humans, but the proportion we observed was higher overall. We discount the possibility of major sample contamination, as negative controls did not produce a positive result and no sequenced samples matched the positive control. These mosquitoes fed on humans from April to August, when the zoos have more visitors and temporary staff, and no human blood meals were found at other times, although the occurrence of birds and other mammalian blood meals continued to be observed. Additionally, the choice for humans was significantly higher in *Cx. pipiens* than in *Cs. annulata*, which is unexpected, as *Cs. annulata* has been reported as a biting nuisance for people in the UK [53]. PCR identification showed that all *Cx. pipiens* that fed on humans belonged to the *pipiens* biotype, as this biotype is typically described as ornithophilic; abundance of visitors and staff seem to be a relevant influence in mosquito feeding patterns in zoos.

*Anopheles maculipennis* s.l. prefers to feed on mammals over birds [7, 54]. However, two mosquitoes from this group fed on Humboldt penguins (*Spheniscus humboldti*), which to our knowledge is the first report of this host choice. Due to the low sample size, we cannot conclude whether there is a host pattern or simply a tendency of capturing blood-fed mosquitoes in proximity to the vertebrates they feed on, as these mosquitoes were captured close to the penguin exhibit (<22m). Therefore, targeted sampling of this group is needed and should include the molecular identification of the species, as they have different host patterns [54].

A correlation between mosquito abundance and proportion of blood-feds has been noticed before [11]. During the first year in Chester Zoo, we found high mosquito abundance and high proportion of blood-feds in areas A1 and A3 and again for A1 in 2018. In Flamingo Land, we observed a similar situation in area A2. Nevertheless, other areas with lower general catches showed high proportions of blood-fed mosquitoes, like A7 and A10 in Chester Zoo in 2017 and areas A10 and A11 in 2018. Therefore, the mosquito abundance is not the only explanatory factor. For instance, area A3 in Chester Zoo captured a high number of blood-fed mosquitoes in 2017 possibly due to its proximity to a picnic area and a childrens playground. Fewer mosquitoes were caught in this area in 2018, when the childrens playground was closed for renewal over several weeks, and the off-show aviaries next to the sampling area were expanded, reducing a considerable portion of the vegetation. In Chester Zoo, area A7, which caught more blood-feds than expected in 2017, is also near a picnic garden and an area with high transit of visitors. In 2017 and 2018, area A10 also captured a high proportion of blood-feds which matched mainly wild birds. This area is inside a wetland aviary where several species are kept, and the abundance of wild passerines could be high, as they are attracted to the waterfowls food despite the netting of the exhibit. In Flamingo Land, area A2, also with a higher proportion of blood-feds, is in the boundary of the South American exhibit which contains large mammals such as capybara (*Hydrochoerus hydrochaeris*), on which we found two mosquitoes feeding. It appears that the constant presence of suitable hosts is an attractant factor for mosquitoes.

The mosquito host feeding patterns varied within each year. There is evidence that host selection by *Culex* spp. is influenced primarily by the availability of preferred hosts [55]; therefore, these variations possibly depend on the mosquito abundance and host availability, both of which increase during the summer. We observed a significant host shift in the case of *Cx. pipiens* in Chester Zoo in 2017, preferring humans in June and birds in July. Tuten et al. [14] also observed a host shift for *Cx. pipiens*, although in this case preferring birds in the summer and birds and mammals in autumn. These changes could be related to host availability influenced, for instance, by the migration and breeding seasons of birds (nestlings are more prone to mosquito bites [8]). Furthermore, when the preferred hosts of *Culex* spp. are scarce, this mosquito can shift to other hosts, including humans [25].

Some species like *Cx. pipiens* breed and rest close to their hosts habitat [10], so it is reasonable that the chances of capturing blood-fed mosquitoes are higher closer to the location of potential hosts, as we observed for zoo vertebrates: one third of the mosquitoes that fed on zoo vertebrates (*n*=9) were captured within 50m from the exhibits where they had fed and more than half (*n*=15) within 100m. Other authors reported maximum travelling distances of between 170 and 770m [8, 14, 15, 56], and the maximum that we observed was within this range (367m). However, we found mosquitoes feeding on domestic animals (cattle, pig, chicken, and dog) that we assume came from nearby farms or dwellings. Thus, dispersal was probably further than our estimate.

It is possible that in some areas the landscape forms flight paths that aid mosquitos movement in a certain direction [56], and the dominant wind direction could be a relevant factor influencing dispersal and flight direction [57]. For example, area A1 in Chester Zoo captured blood-fed mosquitoes that feed on zoo vertebrates located from the southwest to the northwest from this area, and the wind on the day before collection came from a similar direction in the case of four out of six mosquitoes. The use of portable weather stations could improve the study of wind and landscape influence in mosquito dispersal. It is important to consider that using the exhibits centroid, or any other measurement to the exhibit, assumes that the vertebrates distribute randomly within their exhibits, when in reality they spend more time in certain areas, like around the feeders during the day or in the enclosure at night-time. The estimation of animal occupancy degrees inside the exhibits should be considered for a more precise assessment of mosquito travelling distances.

Temperature affects mosquito feeding activity, reproduction, longevity, and development [58, 59], which could explain the positive relationship with the capture of blood-fed mosquitoes shown in the models. High relative humidity may enhance the effect of odorant cues for host-seeking mosquitoes [25]; however, our models showed mostly a negative relation, possibly because higher humidity promotes mosquito dispersal [10] and thus decreases the chances of capturing them. Precipitation, on the other hand, significantly increased the chances of capturing blood-fed mosquitoes according to three models; this is counterintuitive, as mosquitoes are not expected to fly under rainy conditions. However, this variable was aggregated weekly over the sampling units; thus, higher values do not imply more constant rain but more rain over a weeks time. Therefore, rainfall may reduce mosquito dispersal, increasing the capture chances, but does not prevent their feeding activity. Both Brugman et al. [7] and Karki et al. [57] reported that wind diminishes the capture of blood-fed mosquitoes, which can be explained by mosquitoes taking shelter under windy conditions, thus reducing the host-seeking activity and the dispersal after blood feeding. Wind speed was significant in two models but in opposite directions, so we cannot conclude its influence in our samplings. In one model, scarce vegetation was associated with an increase in blood-fed mosquito capture, suggesting that mosquitoes that are looking for shelter are attracted to the traps in the absence of natural resting areas. Finally, a close distance to zoo vertebrate exhibits significantly increased the likelihood of capturing blood-fed mosquitoes in one model, possibly due to dispersal behaviour as discussed before in relation to travelling distances.

The high proportion of *Cx. pipiens* mosquitoes feeding on humans that we observed represents not only a likely nuisance for visitors and staff at the zoos, but also a potential risk for disease transmission. *Culex* spp. are vectors of viruses hosted by wild birds, such as West Nile virus (WNV), Sindbis virus (SINV), and Usutu virus (USUV), all of which have been reported circulating in mainland Europe [29, 60] and could pose a serious threat if they are introduced to the UK. Moreover, all mixed blood meals that we found were from *Cx. pipiens* involving a bird host, and the mixed blood meals that included humans were combined with blood of Eurasian magpie (*Pica pica*), which is a proven natural reservoir of WNV and an effective target for its surveillance [61]. In addition, it has been shown that the temporal and spatial variation in host preferences by *Culex* spp. can influence the timing and severity of WNV infections, probably in relation to its seasonal shifts between ornithophilic and anthropophilic cycles [11, 62]. Although the host shift in *Cx. pipiens* that we observed in one of our samplings occurred from humans to birds, the potential of this mosquito as a bridge vector for humans and domestic animals (i.e. horses) [48, 63] should be constantly monitored, despite the lack of evidence confirming the establishment of WNV in the UK [29, 64], as well as for the other mentioned viruses, especially after the confirmed presence of USUV in the UK [22].

The interspecific transmission of vector-borne diseases is also important for the health of the animals in the zoo collections. For instance, mosquitoes have been involved in the transmission of Eastern equine encephalitis virus to African penguins (*Spheniscus demersus*) in North America, USUV to great grey owls (*Strix nebulosa*) in Austria, and WNV that has caused the death of exotic animals in roughly 100 zoos in the United States [13]. The risk of disease transmission between local bird species and zoo vertebrates is present in our study sites, as avian malaria has caused outbreaks in Humboldt penguin (*Spheniscus humboldti*) colonies in both Chester Zoo and Flamingo Land [19]. Interestingly, host preference changes can also influence the transmission dynamics of avian malaria parasites in bird communities [55]. We found four mosquitoes feeding on Humboldt penguins, two *Anopheles maculipennis* s.l., one *Cx. pipiens*, and an unknown Culicinae, which was likely *Cx. pipiens*, as it had all the corresponding morphological features except for those evaluated on the legs. *Anopheles* spp. mosquitoes are considered potential vectors for avian *Plasmodium* spp. and have been found susceptible to the parasite infection experimentally [65]. Therefore, this genus could have a relevant role in the transmission of avian malaria, although *An. maculipennis* s.l. has not been found infected with avian *Plasmodium* yet [49, 66]. To clarify the host patterns of this mosquito genus and *Plasmodium* spp. transmission risks, the precise identification of its species is needed.

## Conclusions

Zoological gardens provide unique opportunities for the study of mosquitoes; thus, abundance, host choice, and dispersal can be explored to assess disease transmission risks. We confirmed that mosquitoes in zoos conserve their characteristic host patterns to a certain degree and feed on a wide range of hosts, and that they presented an important and unexpected choice for humans which varied between 10 and 63% in our samplings. This highlights the risk of zoonotic viruses transmitted between humans and birds such as WNV, USUV, and SINV if ever established in the UK. There is an implied health risk for zoo birds, as we found mosquitoes feeding on them. Of especial concern are the Javan green magpies (*Cissa thalassina*), which are critically endangered, and the Humboldt penguins (*Spheniscus humboldti*), due to their high susceptibility to avian malaria. Mosquito feeding behaviour is influenced by different factors and it changes temporally and spatially. In our samplings, the main period of feeding activity varied with year and location, it corresponded with the overall increase in mosquito abundance, and it was mainly influenced by temperature in a positive sense. We captured a high proportion of blood-fed mosquitoes in areas with high captures of mosquitoes in general, but this was not always the case. Mosquito dispersal after a blood meal was variable, and it is likely that landscape features influence their movements. Therefore, mosquito feeding behaviour is affected by weather, landscape, and abundance of potential hosts. Our results highlight the complexity of mosquito ecology in zoos and the relevance of assessing the risk of interspecific transmission of pathogens, which need to be thoroughly understood for the efficient control of mosquito populations and reduction of vector-host contact in zoological gardens.

## Supplementary Information

**Additional file1: Table S1.** Variables and values considered for the environmental analysis. **Table S2.** Values of the categorical variables for each trap. **Table S3.** Host choices of blood-fed mosquitoes captured in Chester Zoo, 2017. **Table S4.** Host choices of blood-fed mosquitoes captured in Chester Zoo, 2018. **Table S5.** Host choices of blood-fed mosquitoes captured in Chester Zoo, 2019. **Table S6.** Host choices of blood-fed mosquitoes captured in Flamingo Land, 2017. **Table S7.** Values of the generalised linear models for the significant variables in relation to the capture of blood-fed mosquitoes. **Figure S1.** Host patterns of blood-fed mosquitoes. Birds in shades of blue, non-human mammals in shades of red. Zoo vertebrates are indicated with an asterisk.

## Data Availability

All data relevant for this study are contained in this published article and the Additional files. The datasets for this study are available from the corresponding author on reasonable request.

## References

[1] Abella-Medrano CA, Ibanez-Bernal S, Carbo-Ramirez P, Santiago-Alarcon D (2018). Blood-meal preferences and avian malaria detection in mosquitoes (Diptera: Culicidae) captured at different land use types within a neotropical montane cloud forest matrix. Parasitol Int.

[2] European Centre for Disease Prevention and Control (2012). Guidelines for the surveillance of invasive mosquitoes in Europe.

[3] Cebrian-Camison S, Puente JML, Figuerola J (2020). A literature review of host feeding patterns of invasive Aedes mosquitoes in Europe. Insects.

[4] Metelmann S, Caminade C, Jones AE, Medlock JM, Baylis M, Morse AP (2019). The UKs suitability for *Aedes albopictus* in current and future climates. J R Soc Interface.

[5] Kramer LD, Ciota AT (2015). Dissecting vectorial capacity for mosquito-borne viruses. Curr Opin Virol.

[6] Dye C (1986). Vectorial capacity: must we measure all its components?. Parasitol Today.

[7] Brugman VA, Hernandez-Triana LM, England ME, Medlock JM, Mertens PP, Logan JG (2017). Blood-feeding patterns of native mosquitoes and insights into their potential role as pathogen vectors in the Thames estuary region of the United Kingdom. Parasites Vectors.

[8] Heym EC, Kampen H, Schafer M, Walther D (2019). Mosquito bloodmeal preferences in two zoological gardens in Germany. Med Vet Entomol.

[9] Santos CS, Pie MR, da Rocha TC, Navarro-Silva MA (2019). Molecular identification of blood meals in mosquitoes (Diptera, Culicidae) in urban and forested habitats in southern Brazil. PLoS ONE.

[10] Becker N, Petric D, Zgomba M, Boase C, Dahl C, Madon M (2010). Mosquitoes and their control.

[11] Goodman H, Egizi A, Fonseca DM, Leisnham PT, LaDeau SL (2018). Primary blood-hosts of mosquitoes are influenced by social and ecological conditions in a complex urban landscape. Parasites Vectors.

[12] Orsborne J, Furuya-Kanamori L, Jeffries CL, Kristan M, Mohammed AR, Afrane YA (2019). Investigating the blood-host plasticity and dispersal of *Anopheles coluzzii* using a novel field-based methodology. Parasites Vectors.

[13] Adler PH, Tuten HC, Nelder MP (2011). Arthropods of medicoveterinary importance in zoos. Annu Rev Entomol.

[14] Tuten HC, Bridges WC, Paul KS, Adler PH (2012). Blood-feeding ecology of mosquitoes in zoos. Med Vet Entomol.

[15] Greenberg JA, DiMenna MA, Hanelt B, Hofkin BV (2012). Analysis of post-blood meal flight distances in mosquitoes utilizing zoo animal blood meals. J Vector Ecol.

[16] Ludwig GV, Calle PP, Mangiafico JA, Raphael BL, Danner DK, Hile JA (2002). An outbreak of West Nile virus in a New York City captive wildlife population. Am J Trop Med Hyg.

[17] Silveira P, Belo NO, Lacorte GA, Kolesnikovas CK, Vanstreels RE, Steindel M (2013). Parasitological and new molecular-phylogenetic characterization of the malaria parasite *Plasmodium tejerai* in South American penguins. Parasitol Int.

[18] Grilo ML, Vanstreels RE, Wallace R, Garcia-Parraga D, Braga EM, Chitty J (2016). Malaria in penguins: current perceptions. Avian Pathol.

[19] Hernandez-Colina A. Ecology of mosquito vectors in relation to avian malaria in zoological gardens in the United Kingdom. Ph.D. thesis. Liverpool: The University of Liverpool; 2019.

[20] Rijks J, Kik M, Slaterus R, Foppen R, Stroo A, IJzer J (2016). Widespread *Usutu virus* outbreak in birds in the Netherlands 2016. Euro Surveill.

[21] Buchebner N, Zenker W, Wenker C, Steinmetz HW, Ss E, Lussy H (2013). Low *Usutu virus* seroprevalence in four zoological gardens in central Europe. BMC Vet Res.

[22] Folly AJ, Lawson B, Lean FZ, McCracken F, Spiro S, John SK (2020). Detection of *Usutu virus* infection in wild birds in the United Kingdom, 2020. Euro Surveill.

[23] Human Animal Infections and Risk Surveillance (HAIRS) Group (2020). Qualitative assessment of the risk that Usutu virus presents to the UK human population.

[24] Stephenson EB, Murphy AK, Jansen CC, Peel AJ, McCallum H (2019). Interpreting mosquito feeding patterns in Australia through an ecological lens: an analysis of blood meal studies. Parasites Vectors.

[25] Takken W, Verhulst NO (2013). Host preferences of blood-feeding mosquitoes. Annu Rev Entomol.

[26] Ciota AT, Kramer LD (2013). Vector-virus interactions and transmission dynamics of West Nile virus. Viruses.

[27] Farajollahi A, Fonseca DM, Kramer LD, Marm KA (2011). Bird biting mosquitoes and human disease: a review of the role of *Culex pipiens* complex mosquitoes in epidemiology. Infect Genet Evol.

[28] Pinheiro RB, Felix GM, Chaves AV, Lacorte GA, Santos FR, Braga EM (2016). Trade-offs and resource breadth processes as drivers of performance and specificity in a host-parasite system: a new integrative hypothesis. Int J Parasitol.

[29] Folly A, Dorey-Robinson D, Hernndez-Triana L, Phipps L, Johnson N (2020). Emerging threats to animals in the United Kingdom by arthropod-borne diseases. Front Vet Sci.

[30] Schonenberger AC, Wagner S, Tuten HC, Schaffner F, Torgerson P, Furrer S (2016). Host preferences in host-seeking and blood-fed mosquitoes in Switzerland. Med Vet Entomol.

[31] Faust CL, Dobson AP, Gottdenker N, Bloomfield LSP, McCallum HI, Gillespie TR (2017). Null expectations for disease dynamics in shrinking habitat: dilution or amplification?. Philos Trans R Soc Lond B Biol Sci.

[32] Biogents AG. BG-Mosquitaire against tiger mosquitoes. 2017. http://www.biogents.com/bg-mosquitaire/. Accessed 03 Jan 2017.

[33] John W. Hock Company. CDC gravid trap. 2013. http://johnwhock.com/products/mosquito-sandfly-traps/cdc-gravid-trap/. Accessed 03 Jan 2017.

[34] Reiter P (1983). A portable, battery-powered trap for collecting gravid *Culex* mosquitos. Mosq News.

[35] Roiz D, Roussel M, Munoz J, Ruiz S, Soriguer R, Figuerola J (2012). Efficacy of mosquito traps for collecting potential West Nile mosquito vectors in a natural Mediterranean wetland. Am J Trop Med.

[36] John W. Hock Company. Modified CDC backpack aspirator. 2017. https://www.johnwhock.com/products/aspirators/modified-cdc-backpack-aspirator/. Accessed 13 Jan 2017.

[37] Cranston P, Ramsdale C, Snow K, White G (1987). Adults, larvae and pupae of British mosquitoes (Culicidae): a key. Sci Publ No. 48.

[38] Reeves LE, Holderman CJ, Gillett-Kaufman JL, Kawahara AY, Kaufman PE (2016). Maintenance of host DNA integrity in field-preserved mosquito (Diptera: Culicidae) blood meals for identification by DNA barcoding. Parasites Vectors.

[39] Detinova TS (1962). Age-grouping methods in Diptera of medical importance with special reference to some vectors of malaria. Monogr Ser World Health Organ.

[40] Alcaide M, Rico C, Ruiz S, Soriguer R, Munoz J, Figuerola J (2009). Disentangling vector-borne transmission networks: a universal DNA barcoding method to identify vertebrate hosts from arthropod bloodmeals. PLoS ONE.

[41] Hesson JC, Lundstrom JO, Halvarsson P, Erixon P, Collado A (2010). A sensitive and reliable restriction enzyme assay to distinguish between the mosquitoes *Culex torrentium* and *Culex pipiens*. Med Vet Entomol.

[42] Martinez-de la Puente J, Ferraguti M, Ruiz S, Roiz D, Soriguer RC, Figuerola J (2016). *Culex pipiens* forms and urbanization: effects on blood feeding sources and transmission of avian *Plasmodium*. Malar J..

[43] Becker N, Jost A, Weitzel T (2012). The *Culex pipiens* complex in Europe. J Am Mosq Control Assoc.

[44] Bahnck CM, Fonseca DM (2006). Rapid assay to identify the two genetic forms of *Culex* (*Culex*) *pipiens* L. (Diptera: Culicidae) and hybrid populations. Am J Trop Med Hyg.

[45] Species360. Zoological Information Management Software (ZIMS). 2019. https://www.species360.org/products-services/zoo-aquarium-animal-management-software/. Accessed 13 Aug 2019.

[46] R Core Team. R: a language and environment for statistical computing. 3.0.2 ed. Vienna, Austria: R Foundation for Statistical Computing; 2012.

[47] Egizi A, Martinsen ES, Vuong H, Zimmerman KI, Faraji A, Fonseca DM (2018). Using bloodmeal analysis to assess disease risk to wildlife at the new northern limit of a mosquito species. EcoHealth.

[48] Chapman GE, Archer D, Torr S, Solomon T, Baylis M (2017). Potential vectors of equine arboviruses in the UK. Vet Rec.

[49] Martinez-de la Puente J, Munoz J, Capelli G, Montarsi F, Soriguer R, Arnoldi D (2015). Avian malaria parasites in the last supper: identifying encounters between parasites and the invasive Asian mosquito tiger and native mosquito species in Italy. Malar J.

[50] Rizzoli A, Bolzoni L, Chadwick EA, Capelli G, Montarsi F, Grisenti M (2015). Understanding West Nile virus ecology in Europe: Culex pipiens host feeding preference in a hotspot of virus emergence. Parasites Vectors.

[51] Hesson JC, Schafer M, Lundstrom JO (2016). First report on human-biting *Culex pipiens* in Sweden. Parasites Vectors.

[52] Brstler J, Jst H, Garms R, Krger A, Tannich E, Becker N (2016). Host-feeding patterns of mosquito species in Germany. Parasites Vectors.

[53] Asgharian H, Chang PL, Lysenkov S, Scobeyeva VA, Reisen WK, Nuzhdin SV (2015). Evolutionary genomics of *Culex pipiens*: global and local adaptations associated with climate, life-history traits and anthropogenic factors. Proc Biol Sci.

[54] Danabalan R, Monaghan MT, Ponsonby DJ, Linton YM (2014). Occurrence and host preferences of *Anopheles maculipennis* group mosquitoes in England and Wales. Med Vet Entomol.

[55] Kim KS, Tsuda Y (2010). Seasonal changes in the feeding pattern of *Culex pipiens pallens* govern the transmission dynamics of multiple lineages of avian malaria parasites in Japanese wild bird community. Mol Ecol.

[56] Ejiri H, Sato Y, Kim KS, Hara T, Tsuda Y, Imura T (2011). Entomological study on transmission of avian malaria parasites in a zoological garden in Japan: bloodmeal identification and detection of avian malaria parasite DNA from blood-fed mosquitoes. J Med Entomol.

[57] Karki S, Hamer GL, Anderson TK, Goldberg TL, Kitron UD, Krebs BL (2016). Effect of trapping methods, weather, and landscape on estimates of the *Culex* vector mosquito abundance. Environ Health Insights.

[58] Ciota AT, Matacchiero AC, Kilpatrick AM, Kramer LD (2014). The effect of temperature on life history traits of *Culex* mosquitoes. J Med Entomol.

[59] Ewing DA, Cobbold CA, Purse BV, Nunn MA, White SM (2016). Modelling the effect of temperature on the seasonal population dynamics of temperate mosquitoes. J Theor Biol.

[60] Hesson JC, Verner-Carlsson J, Larsson A, Ahmed R, Lundkvist A, Lundstrom JO (2015). *Culex torrentium* mosquito role as major enzootic vector defined by rate of Sindbis virus infection, Sweden, 2009. Emerg Infect Dis.

[61] Napp S, Montalvo T, Piol-Baena C, Gmez-Martn MB, Nicols-Francisco O, Soler M (2019). Usefulness of Eurasian magpies (*Pica pica*) for West Nile virus surveillance in non-endemic and endemic situations. Viruses.

[62] Kilpatrick AM, Kramer LD, Jones MJ, Marra PP, Daszak P (2006). West Nile virus epidemics in North America are driven by shifts in mosquito feeding behavior. PLoS Biol.

[63] Hamer GL, Kitron UD, Brawn JD, Loss SR, Ruiz MO, Goldberg TL (2008). *Culex pipiens* (Diptera: Culicidae): a bridge vector of West Nile virus to humans. J Med Entomol.

[64] Vaux AG, Gibson G, Hernandez-Triana LM, Cheke RA, McCracken F, Jeffries CL (2015). Enhanced West Nile virus surveillance in the North Kent marshes. UK Parasit Vectors.

[65] Huff CG (1965). Susceptibility of mosquitoes to avian malaria. Exp Parasitol.

[66] Inci A, Yildirim A, Njabo KY, Duzlu O, Biskin Z, Ciloglu A (2012). Detection and molecular characterization of avian *Plasmodium* from mosquitoes in central Turkey. Vet Parasitol.

