# Supplementary material for: Blood-feeding ecology of mosquitoes in two zoological gardens in the United Kingdom
Source: Parasit Vectors. 2021 May 21;14:249. doi: 10.1186/s13071-021-04735-0 (PMC8139098; doi:10.1186/s13071-021-04735-0)

**Additional file 1**

**Additional Tables**

**Table S1** Variables and values considered for the environmental analysis

| **Variable** | **Values** |
| --- | --- |
| Vegetation | *Scarce:* few plants no higher than 2m and without trees  *Medium:* some plants like bushes 2m high and lower plants with trees  *Dense:* abundant plants with dense foliage at all levels including tree cover |
| Distance to oviposition sites | *Close:* less than 20m from a suitable oviposition site  *Intermediate:* between 20 and 50m from a suitable oviposition site  *Remote:* more than 50m apart from a suitable oviposition site |
| Resting areas (not considering vegetation) | *Rare:* few unsuitable resting structures, like fences  *Medium:* some resting places available, like walls or containers  *Abundant:* diverse resting places available including sheds and accessible buildings |
| Distance to zoo animal exhibits | *Close:* less than 20m from an open animal exhibit  *Intermediate:* between 20 and 50m from an open animal exhibit  *Remote:* more than 50m apart from an open animal exhibit |
| Regional temperature | Obtained from the closest meteorological station |
| Precipitation | Obtained from the closest meteorological station |
| Local temperature | Recorded with loggers next to the traps |
| Humidity | Recorded with loggers next to the traps |

**Table S2** Values of the categorical variables for each trap

| **Zoo** | **Trap** | **Vegetation** | **Oviposition sites** | **Resting Areas** | **Animals Exhibits** |
| --- | --- | --- | --- | --- | --- |
| **Chester Zoo** | M1 | Medium | Close | Abundant | Close |
|  | M2 | Scarce | Remote | Medium | Close |
|  | M3 | Dense | Intermediate | Rare | Close |
|  | M4 | Scarce | Close | Medium | Intermediate |
|  | M5 | Dense | Remote | Medium | Remote |
|  | M6 | Medium | Intermediate | Medium | Remote |
|  | M7 | Scarce | Remote | Medium | Close |
|  | M10 | Medium | Intermediate | Medium | Close |
|  | M11 | Medium | Intermediate | Rare | Remote |
|  | M12 | Scarce | Remote | Abundant | Close |
|  | M13 | Scarce | Close | Rare | Close |
|  | G1 | Dense | Remote | Medium | Remote |
|  | G2 | Dense | Remote | Medium | Intermediate |
|  | G3 | Medium | Close | Rare | Intermediate |
|  | G4 | Medium | Close | Medium | Intermediate |
|  | G5 | Dense | Remote | Rare | Remote |
|  | G6 | Medium | Intermediate | Medium | Remote |
|  | G7 | Scarce | Remote | Medium | Intermediate |
|  | G10 | Medium | Intermediate | Medium | Close |
|  | G11 | Dense | Intermediate | Rare | Remote |
|  | G12 | Dense | Remote | Medium | Intermediate |
|  | G13 | Scarce | Close | Rare | Close |
| **Flamingo Land** | M1 | Scarce | Remote | Abundant | Close |
|  | M2 | Medium | Remote | Rare | Close |
|  | M3 | Scarce | Remote | Medium | Intermediate |
|  | M4 | Scarce | Remote | Medium | Intermediate |
|  | G1 | Scarce | Remote | Abundant | Close |
|  | G2 | Medium | Remote | Medium | Close |
|  | G3 | Scarce | Close | Medium | Intermediate |
|  | G4 | Medium | Remote | Medium | Remote |
| M: BG-Mosquitaire trap, G: CDC-Gravid trap. The trap numbers correspond to the sampling areas | | | | | |

**Table S3** Host choices of blood-fed mosquitoes captured in Chester Zoo, 2017

| **Host Group** | **Scientific Name** | **Common name** | **Population^a^** | ***Cx. pipiens* (%)** | ***Cs. annulata* (%)** | **Unknown^b^ (%)** | **Total (%)** |
| --- | --- | --- | --- | --- | --- | --- | --- |
| Birds  (n = 26) | *Anas platyrhynchos* | Mallard | - | 1 (1.3) | 0 (0.0) | 0 (0.0) | 1 (1.3) |
|  | *Cissa thalassina* | Javan green magpie^c^ | 14 | 2 (2.7) | 0 (0.0) | 0 (0.0) | 2 (2.7) |
|  | *Corvus monedula* | Eurasian jackdaw | - | 1 (1.3) | 0 (0.0) | 0 (0.0) | 1 (1.3) |
|  | *Passer domesticus* | House sparrow | - | 3 (4.0) | 0 (0.0) | 0 (0.0) | 3 (4.0) |
|  | *Pica pica* | Eurasian magpie | - | 4 (5.3) | 1 (1.3) | 1 (1.3) | 6 (8.0) |
|  | *Prunella modularis* | Dunnock | - | 1 (1.3) | 0 (0.0) | 0 (0.0) | 1 (1.3) |
|  | *Pyrrhula pyrrhula* | Eurasian bullfinch | - | 1 (1.3) | 0 (0.0) | 0 (0.0) | 1 (1.3) |
|  | *Tauraco schalowi* | Schalow's turaco^c^ | 2 | 3 (4.0) | 0 (0.0) | 1 (1.3) | 4 (5.3) |
|  | *Turdus merula* | Eurasian blackbird | - | 5 (6.7) | 0 (0.0) | 1 (1.3) | 6 (8.0) |
|  | *Turdus philomelos* | Song thrush | - | 1 (1.3) | 0 (0.0) | 0 (0.0) | 1 (1.3) |
| Mammals  (n = 7) | *Bos taurus* | Cattle | - | 0 (0.0) | 1 (1.3) | 0 (0.0) | 1 (1.3) |
|  | *Camelus bactrianus* | Bactrian camel^c^ | 2 | 0 (0.0) | 3 (4.0) | 0 (0.0) | 3 (4.0) |
|  | *Rucervus eldii thamin* | Eld's deer^c^ | 10 | 0 (0.0) | 2 (2.7) | 0 (0.0) | 2 (2.7) |
|  | *Tragelaphus eurycerus* | Bongo^c^ | 3 | 0 (0.0) | 1 (1.3) | 0 (0.0) | 1 (1.3) |
| Humans  (n = 37) | *Homo sapiens* | Human | 5380^d^ | 27 (36.0) | 9 (12.0) | 1 (1.3) | 37 (49.3) |
| Mixed blood-meals  (n = 5) | *Camelus bactrianus* / *Turdus merula* | Bactrian camel^c^ / Eurasian blackbird | 2/- | 1 (1.3) | 0 (0.0) | 0 (0.0) | 1 (1.3) |
|  | *Giraffa camelopardalis rothschildi* /  *Tauraco schalowi* | Rothschild's giraffe^c^/ Schalow's turaco^c^ | 10/2 | 1 (1.3) | 0 (0.0) | 0 (0.0) | 1 (1.3) |
|  | *Pica pica* /  *Homo sapiens* | Eurasian magpie / Human | -/5380^d^ | 3 (4.0) | 0 (0.0) | 0 (0.0) | 3 (4.0) |
|  |  | **Total** |  | **54 (72.0)** | **17 (22.7)** | **4 (5.3)** | **75 (100.0)** |
| ^a^: Vertebrate populations during the sampling; ^b^: These mosquitoes could not be identified beyond the Culicinae subfamily due to the absence of legs; ^c^: zoo vertebrates; ^d^: average number of visitors per day during the sampling | | | | | | | |

**Table S4** Host choices of blood-fed mosquitoes captured in Chester Zoo, 2018

| **Host group** | **Scientific Name** | **Common name** | **Population^a^** | ***Cx. pipiens* (%)** | ***Culiseta* spp. (%)** | ***Anopheles maculipennis s.l.* (%)** | **Unknown^b^ (%)** | **Total (%)** |
| --- | --- | --- | --- | --- | --- | --- | --- | --- |
| Birds  (n = 22) | *Anas platyrhynchos* | Mallard | - | 4 (8.9) | 2 (4.4) | 0 (0.0) | 0 (0.0) | 6 (13.3) |
|  | *Gallus gallus* | Chicken | - | 2 (4.4) | 1 (2.2) | 0 (0.0) | 1 (2.2) | 4 (8.9) |
|  | *Cyanistes caeruleus* | Blue tit | - | 3 (6.7) | 0 (0.0) | 0 (0.0) | 0 (0.0) | 3 (6.7) |
|  | *Passer domesticus* | House sparrow | - | 2 (4.4) | 0 (0.0) | 0 (0.0) | 0 (0.0) | 2 (4.4) |
|  | *Spheniscus humboldti* | Humboldt penguin^c^ | 45 | 0 (0.0) | 0 (0.0) | 1 (2.2) | 1 (2.2) | 2 (4.4) |
|  | *Corvus monedula* | Western jackdaw | - | 1 (2.2) | 0 (0.0) | 0 (0.0) | 0 (0.0) | 1 (2.2) |
|  | *Erithacus rubecula* | European Robin | - | 1 (2.2) | 0 (0.0) | 0 (0.0) | 0 (0.0) | 1 (2.2) |
|  | *Strix leptogrammica* | Brown wood-owl^c^ | 3 | 1 (2.2) | 0 (0.0) | 0 (0.0) | 0 (0.0) | 1 (2.2) |
|  | *Turdus merula* | Eurasian blackbird | - | 1 (2.2) | 0 (0.0) | 0 (0.0) | 0 (0.0) | 1 (2.2) |
|  | *Turdus philomelos* | Song thrush | - | 1 (2.2) | 0 (0.0) | 0 (0.0) | 0 (0.0) | 1 (2.2) |
| Mammals  (n = 5) | *Bos taurus* | Cattle | - | 1 (2.2) | 0 (0.0) | 0 (0.0) | 1 (2.2) | 2 (4.4) |
|  | *Camelus bactrianus* | Bactrian camel^c^ | 2 | 0 (0.0) | 1 (2.2) | 0 (0.0) | 0 (0.0) | 1 (2.2) |
|  | *Rucervus eldi* | Eld's deer^c^ | 8 | 0 (0.0) | 1 (2.2) | 0 (0.0) | 0 (0.0) | 1 (2.2) |
|  | *Sus scofra* | Pig | - | 1 (2.2) | 0 (0.0) | 0 (0.0) | 0 (0.0) | 1 (2.2) |
| Humans  (n = 17) | *Homo sapiens* | Human | 6104^d^ | 17 (37.8) | 0 (0.0) | 0 (0.0) | 0 (0.0) | 17 (37.8) |
| Mixed blood-meals  (n = 1) | *Columba palumbus / Streptopelia decaocto* | Wood pigeon / Eurasian collared dove | -/- | 1 (2.2) | 0 (0.0) | 0 (0.0) | 0 (0.0) | 1 (2.2) |
|  |  | **Total** |  | **36 (80.0)** | **5 (11.1)** | **1 (2.2)** | **3 (6.7)** | **45 (100)** |
| ^a^: Vertebrate populations during the sampling; ^b^: These mosquitoes could not be identified beyond the Culicinae subfamily due to the absence of legs; ^c^: zoo vertebrates; ^d^: average number of visitors per day during the sampling | | | | | | | | |

**Table S5** Host choices of blood-fed mosquitoes captured in Chester Zoo, 2019

| **Host group** | **Scientific Name** | **Common name** | **Population^a^** | ***Cx. pipiens* (%)** | ***An. maculipennis s.l* (%)*.*** | **Total**  **(%)** |
| --- | --- | --- | --- | --- | --- | --- |
| Birds  (n = 18) | *Corvus monedula* | Eurasian jackdaw | - | 10 (50.0) | 0 (0.0) | 10 (50.0) |
|  | *Pyrrhula pyrrhula* | Eurasian bullfinch | - | 2 (10.0) | 0 (0.0) | 2 (10.0) |
|  | *Parus major major* | Great tit | - | 1 (5.0) | 0 (0.0) | 1 (5.0) |
|  | *Cyanistes caeruleus* | Blue tit | - | 1 (5.0) | 0 (0.0) | 1 (5.0) |
|  | *Gallinula chloropus* | Common moorhen | - | 1 (5.0) | 0 (0.0) | 1 (5.0) |
|  | *Phoenicopterus ruber* | Caribbean flamingo^b^ | 113 | 1 (5.0) | 0 (0.0) | 1 (5.0) |
|  | *Spheniscus humboldti* | Humboldt penguin^b^ | 46 | 1 (5.0) | 1 (5.0) | 2 (10.0) |
| Humans  (n = 2) | *Homo sapiens* | Human | 6749^c^ | 1 (5.0) | 1 (5.0) | 2 (10.0) |
|  |  | **Total** |  | **18 (90.0)** | **2 (5.0)** | **20 (100.0)** |
| ^a^: Vertebrate populations during the sampling; ^b^: zoo vertebrates; ^c^: average number of visitors per day during the sampling | | | | | | |

**Table S6** Host choices of blood-fed mosquitoes captured in Flamingo Land, 2017

| **Host Group** | **Scientific Name** | **Common name** | ***Cx. pipiens* (%)** | ***Culiseta* spp. (%)** | **Unknown^a^ (%)** | **Total**  **(%)** |
| --- | --- | --- | --- | --- | --- | --- |
| Birds  (n = 1) | *Parus major* | Great tit | 0 (0.0) | 0 (0.0) | 1 (5.3) | 1 (5.3) |
| Mammals  (n = 6) | *Camelus bactrianus* | Bactrian camel^b^ | 0 (0.0) | 1 (5.3) | 0 (0.0) | 1 (5.3) |
|  | *Canis lupus familiaris* | Dog | 1 (5.3) | 0 (0.0) | 0 (0.0) | 1 (5.3) |
|  | *Hydrochoerus hydrochaeris* | Capybara^b^ | 1 (5.3) | 1 (5.3) | 0 (0.0) | 2 (10.5) |
|  | *Oryx dammah* | Scimitar-horned Oryx^b^ | 0 (0.0) | 1 (5.3) | 1 (5.3) | 2 (10.5) |
| Humans  (n = 12) | *Homo sapiens* | Human | 10 (52.6) | 0 (0.0) | 2 (10.5) | 12 (10.5) |
|  |  | **Total** | **12 (63.2)** | **3 (15.8)** | **4 (21.1)** | **19 (100.0)** |
| ^a^: These mosquitoes could not be identified beyond the Culicinae subfamily due to the absence of legs; ^b^: zoo vertebrates | | | | | | |

**Table S7** Values of the generalised linear models for the significant variables in relation to the capture of blood-fed mosquitoes

| **Variable** | **Chester Zoo 2017** | | **Chester Zoo 2018** | | **Chester Zoo 2019** | | **Flamingo Land 2017** | | **Overall** | |
| --- | --- | --- | --- | --- | --- | --- | --- | --- | --- | --- |
|  | **E** | **P** | **E** | **P** | **E** | **P** | **E** | **P** | **E** | **P** |
| Temperature | -0.080 | <0.001 | 0.280 | <0.001 | 0.161 | 0.073* | 0.643 | <0.001 | 0.200 | <0.001 |
| Humidity | -0.080 | 0.001 | 0.045 | 0.002 | - | - | -0.042 | <0.001 | -0.017 | <0.001 |
| Precipitation | 0.387 | <0.001 | - | - | - | - | 0.296 | 0.001 | 0.207 | <0.001 |
| Wind speed | - | - | - | - | -0.183 | 0.004 | 0.268 | <0.001 | - | - |
| Scarce vegetation | - | - | - | - | 0.794 | 0.003 | - | - | - | - |
| Close distance to animal exhibits | 0.519 | 0.005 | - | - | - | - | - | - | - | - |
| E: estimate; P: P-value; *: close to significance | | | | | | | | | | |

**Additional Figures**

**Fig. S1** Host preferences of blood-fed mosquitoes. Birds in shades of blue, non-human mammals in shades of red. Zoo vertebrates are indicated with an asterisk


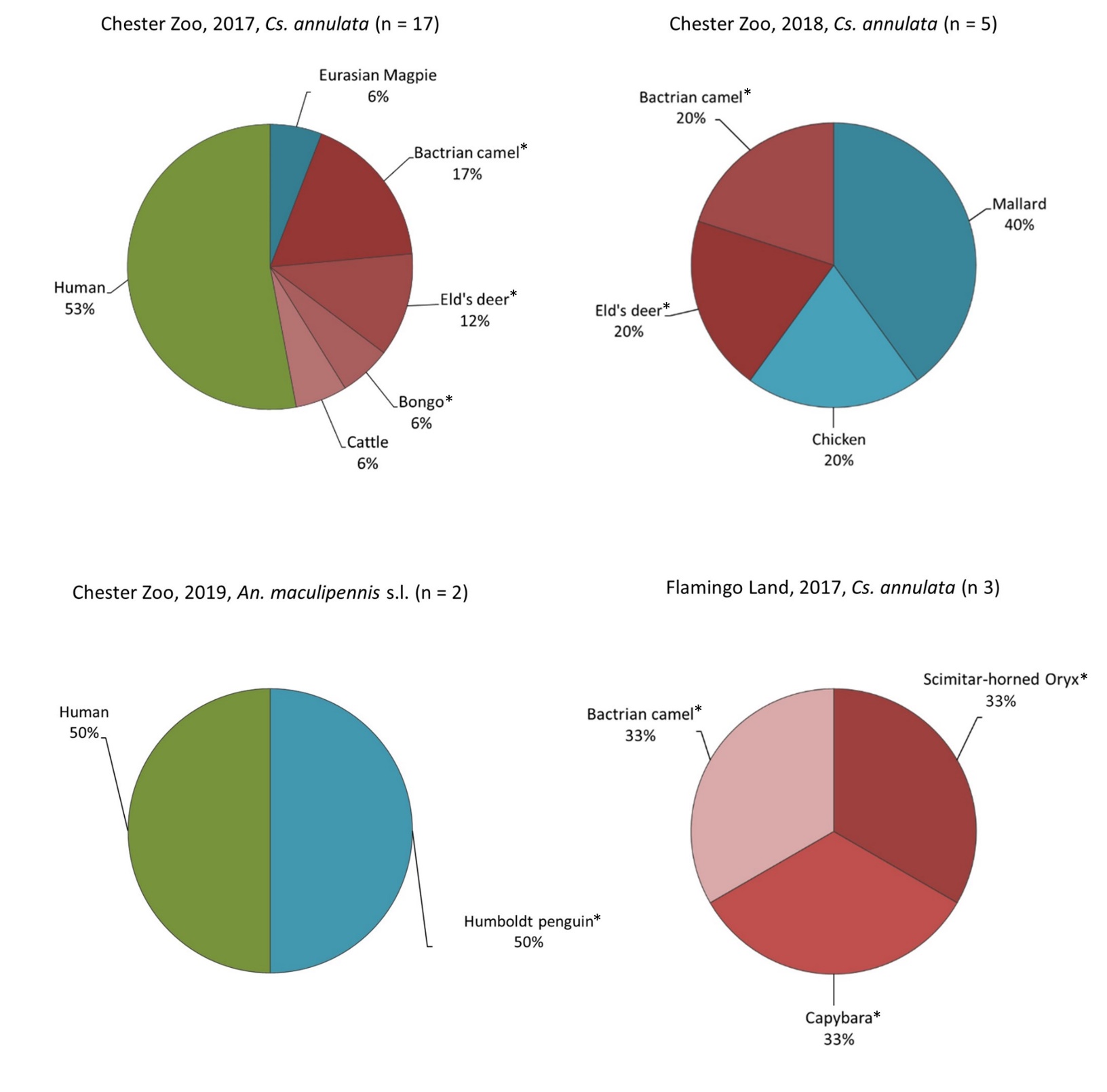

Supplement: Supplementary file 1 — Additional file1: Table S1. Variables and values considered for the environmental analysis. Table S2. Values of the categorical variables for each trap. Table S3. Host choices of blood-fed mosquitoes captured in Chester Zoo, 2017. Table S4. Host choices of blood-fed mosquitoes captured in Chester Zoo, 2018. Table S5. Host choices of blood-fed mosquitoes captured in Chester Zoo, 2019. Table S6. Host choices of blood-fed mosquitoes captured in Flamingo Land, 2017. Table S7. Values of the generalised linear models for the significant variables in relation to the capture of blood-fed mosquitoes. Figure S1. Host patterns of blood-fed mosquitoes. Birds in shades of blue, non-human mammals in shades of red. Zoo vertebrates are indicated with an asterisk. [file 13071_2021_4735_MOESM1_ESM.docx]
